# Actinobacteria From Desert: Diversity and Biotechnological Applications

**DOI:** 10.3389/fmicb.2021.765531

**Published:** 2021-12-09

**Authors:** Feiyang Xie, Wasu Pathom-aree

**Affiliations:** ^1^Doctor of Philosophy Program in Applied Microbiology (International Program), Faculty of Science, Chiang Mai University, under the CMU Presidential Scholarship, Chiang Mai, Thailand; ^2^Research Center of Microbial Diversity and Sustainable Utilization, Department of Biology, Faculty of Science, Chiang Mai University, Chiang Mai, Thailand

**Keywords:** actinobacteria, desert, arid environment, diversity, cultivability, bioactive compounds, natural products, plant growth promoting actinomycetes

## Abstract

Deserts, as an unexplored extreme ecosystem, are known to harbor diverse actinobacteria with biotechnological potential. Both multidrug-resistant (MDR) pathogens and environmental issues have sharply raised the emerging demand for functional actinobacteria. From 2000 to 2021, 129 new species have been continuously reported from 35 deserts worldwide. The two largest numbers are of the members of the genera *Streptomyces* and *Geodermatophilus*, followed by other functional extremophilic strains such as alkaliphiles, halotolerant species, thermophiles, and psychrotolerant species. Improved isolation strategies for the recovery of culturable and unculturable desert actinobacteria are crucial for the exploration of their diversity and offer a better understanding of their survival mechanisms under extreme environmental stresses. The main bioprospecting processes involve isolation of target actinobacteria on selective media and incubation and selection of representatives from isolation plates for further investigations. Bioactive compounds obtained from desert actinobacteria are being continuously explored for their biotechnological potential, especially in medicine. To date, there are more than 50 novel compounds discovered from these gifted actinobacteria with potential antimicrobial activities, including anti-MDR pathogens and anti-inflammatory, antivirus, antifungal, antiallergic, antibacterial, antitumor, and cytotoxic activities. A range of plant growth-promoting abilities of the desert actinobacteria inspired great interest in their agricultural potential. In addition, several degradative, oxidative, and other functional enzymes from desert strains can be applied in the industry and the environment. This review aims to provide a comprehensive overview of desert environments as a remarkable source of diverse actinobacteria while such rich diversity offers an underexplored resource for biotechnological exploitations.

## Introduction

A serious global health problem caused by pathogenic microorganisms is threatening human lives and welfare at an alarming speed. Due to abusive uses of antibiotics and antimicrobial prescriptions, the effectiveness of drugs and vaccines has been sharply decreased. Worse yet, the reduced interest from large pharmaceutical companies has further compromised the drug development process. To date, growing resistant infections have seriously jeopardized several of the United Nations (UN) Sustainable Development Goals (SDGs) related to the economy, global health, food production, and environment (Medina et al., 2020). Infectious diseases, such as malaria, HIV/AIDS, tuberculosis, and other communicable diseases, continue to pose risks to global health. In 2018, more than half a million new cases were caused by multidrug-resistant (MDR) pathogenic infections, which remained the top deadly global disease during the past decade (World Health Organization, 2020). The large-scale use of antibiotics as growth promoters in agriculture also contributed to this crisis. As a result, more than 700,000 antimicrobial resistance-related deaths are predicted to happen annually worldwide in 2050, which could further reduce the global economy by $100 trillion as estimated by UN agencies (Medina et al., 2020). In particular, the pharmaceutical industry was unable to deliver effective drugs during the severe acute respiratory syndrome (SARS) epidemic between 2002 and 2003, causing a fatality rate of 11% (Chan-Yeung and Rui-Heng, 2003). Even today, 17 years later, there is still no anti-SARS drug available to cure the disease due to the slow progress in the development of post-epidemic drugs. Therefore, novel bioactive compounds to combat the threat of MDR pathogens and emerging diseases are urgently needed (Choudhary et al., 2016). Microbial natural products are considered as one of the important sources of bioactive compounds. Natural products are secondary metabolites (SMs) synthesized by bacteria and fungi and are not required for the growth, development, and reproduction of the producing organisms. In their native environments, these SMs may function as signaling molecules (Traxler and Kolter, 2015). SMs are attractive for drug discovery programs, in general, owing to their properties such as biochemical specificity, metabolic selectivity, structural diversity, and the potential to pass through eukaryotic outer membranes to block the production of cellular macromolecules and target enzymes (Baltz, 2008). To date, over 23,000 microbial natural products have been identified since the discovery of penicillin (Katz and Baltz, 2016).

Microorganisms isolated from extreme habitats are considered a significant reservoir for the search and discovery of new drugs. The most recent review revealed that 186 novel compounds were produced by 129 microbes (actinobacteria, cyanobacteria, fungi, and other bacteria) isolated from extreme habitats between 2010 and 2018 (Sayed et al., 2020). Cyanobacteria are known to generate antimicrobial (Micallef et al., 2015), antiprotozoal, and anti-inflammatory compounds (Vijayakumar and Menakha, 2015). Other bacteria reported to potentially synthesize bioactive metabolites are *Thermus filiformis*, which produced four antioxidant carotenoids, namely, all-*trans*-zeaxanthin, thermobiszeaxanthins, thermozeaxanthins, and zeaxanthin monoglucoside (Mandelli et al., 2012). Actinobacteria are shown to be the most frequent producer of specialized metabolites as compared to cyanobacteria and other bacteria (Sayed et al., 2020). The exploration of actinobacteria holds great promise in terms of diverse chemical libraries including antimicrobial compounds (erythromycin, gentamicin, vancomycin, daptomycin, and rifamycin), anticancer compounds (bleomycin, actinomycin D, and mitomycin), immunosuppressive compounds (rapamycin and tacrolimus), antiparasitic compounds (avermectins and spinosyns), and herbicidal compounds (bialaphos and glufosinate) and hence continued to drive innovative biotechnology (Krug and Müller, 2014; Katz and Baltz, 2016; Genilloud, 2017; Jose et al., 2021). Thus, actinobacteria are expected to play a significant role in biotechnological applications including in medical and agricultural sectors.

## Actinobacteria: A Unique Source of Bioactive Compounds

Actinobacteria is one of the largest Gram-positive bacterial groups with high guanine-plus-cytosine (G + C) content (Barka et al., 2016). Many actinobacteria can produce mycelium, known as aerobic filamentous actinobacteria, and further reproduce by sporulation (Barka et al., 2016). Most actinobacteria are free-living microorganisms that can be found in terrestrial or aquatic habitats, including extreme environments such as caves, deep seas, deserts, and mangroves (Macagnan et al., 2006). These ecosystems are proven to be a prolific source of novel actinobacteria (Hong et al., 2009; Abdel-Mageed et al., 2010; Kamjam et al., 2017; Goodfellow et al., 2018; Bull and Goodfellow, 2019; Rangseekaew and Pathom-aree, 2019). Abundant bioactive metabolites have been produced by actinobacteria, such as antibiotics, anticancer drugs, immunosuppressive drugs, enzymes, enzyme inhibitors, and other therapeutic or biologically active compounds (Adeela et al., 2018; Rateb et al., 2018; Law et al., 2020; Almuhayawi et al., 2021; Jose et al., 2021). There are around 589 novel compounds with various chemical structures reported from actinobacteria between 2015 and 2020, more than half of them (52%) with potential bioactivities, such as antiparasitic, anti-quorum sensing, antiviral, and chelating activities (Jose et al., 2021). Remarkably, members of the actinobacterial taxa, including rare actinobacteria, are responsible for the production of over 10,000 pharmaceutical agents (Jose et al., 2021). With respect to aerobic filamentous actinobacteria, the genus *Streptomyces* is an extremely good source of novel specialized metabolites as it was reported to produce 39% of all known microbial metabolites (Sivalingam et al., 2019). To date, members of the genus *Streptomyces* are responsible for about two-thirds of the frontline antibiotics in clinical use, as well as other anticancer, antifungal, and anthelmintic compounds (Law et al., 2019; Al-Dhabi et al., 2020; Ser et al., 2020; Yi et al., 2020). Rare actinobacteria are generally defined as members of actinobacterial taxa that are difficult to isolate from the environment (Amin D. H. et al., 2020). Members of some rare actinobacteria are comparable to their *Streptomyces* neighbors in term of bioactive compound production. These prolific rare actinobacterial species belong to the families of *Micromonosporaceae* (1,061), *Mycobacteriaceae* (483), *Pseudonocardiaceae* (327), *Streptosporangiaceae* (154), and *Thermomonosporaceae* (633) (Selim et al., 2021). Other unclassified species with reported ability to produce bioactive metabolites belonged to the genera of *Actinosporangium* (30), *Alkalomyces* (1), *Catellatopsora* (1), *Elaktomyces* (3), *Erythrosporangium* (1), *Excelsospora* (3), *Frankia* (7), *Kitasatoa* (5), *Microechinospora* (1), *Microellobosporia* (11), *Salinospora* (1), *Sebekia* (3), *Streptoplanospora* (1), *Synnenomyces* (4), *Waksmania* (3), and *Westerdykella* (6) (Selim et al., 2021). Based on the metagenomic analyses and rank abundance analyses, diverse rare actinobacterial taxa are detected in the desert with their population significantly higher (over 34%) than that of validly published taxa (Idris et al., 2017a). This diversity has been poorly recovered by current culture-dependent techniques. The improved isolation method could allow researchers to obtain these abundant rare actinobacteria from deserts for bioprospecting programs.

The genome sizes of actinobacteria ranged from 1 to 12 Mb with potent biosynthetic gene clusters (BGCs) encoded by biologically active compounds (Větrovský and Baldrian, 2013). The term “gifted actinobacteria” is first coined by Richard H. Baltz for actinobacteria with a large genome size, coding for more SM production than other taxa (Baltz, 2017). Quantitative analyses of microbial genomes performed by antiSMASH 3.0 revealed that these gifted actinobacteria harbored 20 to 50 SM gene clusters, allocating 0.8–3.0 Mb of the entire genome to SM production (Baltz, 2017, 2018). For example, gifted strains of *Amycolatopsis*, *Micromonospora*, and *Streptomyces* have moderate (about 5.0–7.9 Mb) or large (over 8 Mb) genomes, harboring 19–20 and >30 BGCs, respectively (Baltz, 2017, 2018, 2019; Nouioui et al., 2019). Whole-genome analysis of these gifted genera usually revealed 20–50 BGCs encoding for both known and/or predicted specialized metabolites, which provides strong evidence that actinobacteria are promising producers of diverse novel compounds with various bioactivities (Bentley et al., 2002; Tang et al., 2016). For example, *Streptomyces coelicolor* A3(2) harbors more than 20 BGCs that encode for specialized metabolites such as blue-colored actinorhodin and red-pigment undecylprodigiosin (Bednarz et al., 2021). Similarly, the genome of *Streptomyces* sp. VN1 has shown diverse known and/or novel metabolites encoded by 34 gene clusters, involving a furan-type anticancer agent (Nguyen et al., 2020).

These genome sequence data also allowed the observation of genomic heterogeneity, which provides great insights into the ability of actinobacteria to synthesize diverse specialized metabolites (Jose et al., 2021). Generally, the genome size may correlate with a more complicated environment, implying that the genome encodes significant metabolic and stress-tolerant properties (Baltz, 2018). For example, *Jiangella gansuensis* YIM 002^T^ isolated from Gansu Desert has a complete genome sequence that identified 45 biosynthetic clusters, suggesting it has high capacity to produce SMs. Compatible solutes, ion transporters, nitrite reductase, and nitrogen fixation proteins were also detected (Jiao et al., 2017). In addition, *J. gansuensis* YIM 002^T^ has a smaller genome (5.59 Mb) compared to other *Jiangella* species (more than 7 Mb), yet it contains 2,504 functional proteins, implying that it may have discarded many genes for adaptation in the harsh desert environments through its evolution (Jiao et al., 2017). To defend against desert environmental stresses, superfluous and non-essential genes were discarded to improve survival efficiency, resulting in desert strains with higher metabolic capabilities compared with unevolved strains (Burke and Moran, 2011). Genome rearrangements were promoted by mobile DNA, which recombines inactive genes into functional genes, whereas inactivated genes were removed through deletion (Burke and Moran, 2011). This genomic evolution suggested that the reduction in genomic size of desert strains facilitated long-term adaptive evolution.

## Desert: An Unexplored Reservoir for Novel Actinobacteria

An increasing number of novel actinobacterial taxa continued to be reported from natural habitats, especially extreme biomes under harsh environmental conditions. These extreme habitats represent a potentially rich resource for novel compounds with biological properties. Particularly, the desert is the largest continental ecosystem on earth with approximately 30% of the total land area while 7% of it is a hyper-arid region with water constraints (Neilson et al., 2012). Desert ecosystems have attracted the increasing interest of microbiologists in the quest for novel bioactive compounds since they are considered an unexplored home of new extremophiles with high tolerance to extreme conditions. Desert environmental conditions are characterized by extremely high or low temperatures, lack of nutrients, very low organic carbon sources, high levels of oxidants and UV radiation or pH or salinity, high concentrations of metals, and other abiotic stresses (Horikoshi et al., 2010; Köberl et al., 2011; Bull et al., 2016; Idris et al., 2017a). The most explored desert soil sample has over 92% new actinobacterial species isolated. However, it is worth to note that the remaining 8% of novel species were found from desert plants, rhizosphere soil, and rock surfaces, respectively. Hence, such extreme conditions in the desert are considered as a selective pressure that shapes diverse actinobacterial communities.

**FIGURE 1:**
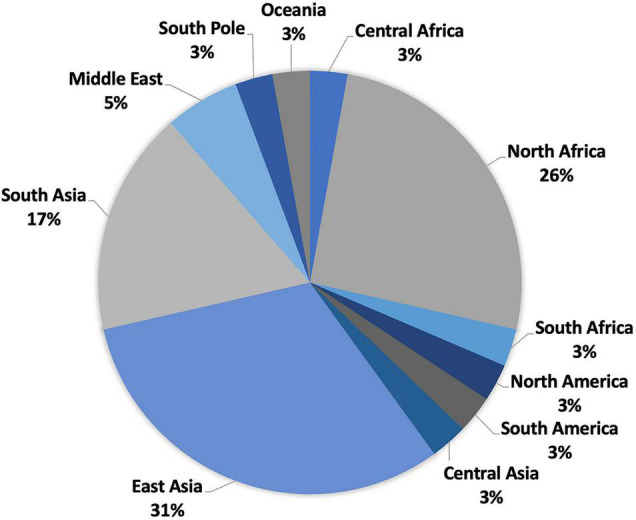
Distribution of desert biomes.

Between 2000 and 2021, numerous diverse newly identified actinobacteria have been recorded from 35 deserts scattered over 11 regions, as summarized in Figure 1 and Supplementary Table 1. These deserts are distributed in the regions of East Asia (31%), Central Asia (3%), Asia (17%), the Middle East (4%), North Africa (26%), South America (3%), Central Africa (3%), North America (3%), South Africa (3%), South Pole (3%), and Oceania (3%).

### Deserts in East Asia

Most deserts (31%) are distributed in China, East Asia (Figure 1 and Supplementary Table 1), including Badain Jaran Desert, Fukang Desert, Gansu Desert, Gurbantunggut Desert, Hangjin Banner Desert, Isolaginsha Desert, Taklamakan Desert, Tengger Desert, Tibet Autonomous Color Desert, Turpan Desert, and Xinjiang Desert. Taklamakan Desert in southwest Xinjiang is home to the greatest number of new species (14). Despite being the largest desert in China, the Taklamakan Desert is the warmest and driest desert in the country, with an annual temperature of around 39°C (Rittner et al., 2016). Additionally, as the second-largest non-polar and sand-shifting desert in the world, the Taklamakan Desert also experiences an extremely cold temperature (−32°C) in winter and an extremely low average annual rainfall ranging from 10 to 38 mm (Rittner et al., 2016). Such severe environmental circumstances have attracted the attention of researchers for exploration of novel actinobacteria with exceptional tolerance in desert environments.

### Deserts in Central and South Asia

Central Asia and South Asia are home to seven deserts, namely, Karakum Desert in Turkmenistan, Central Asia; Cholistan Desert in Pakistan; Kunjam Pass and Lahaul–Spiti Valley, two cold deserts in the Indian Himalayas; the saline desert of Kutch in India; Thal Desert in Pakistan; and Thar Desert in India (Supplementary Table 1). The Karakum Desert, in particular, was the isolation source of the most diverse (six genera) and numerous (11 new species) novel actinobacteria in Central and South Asia. It is one of the largest deserts in the world and the hottest desert in Central Asia (Ghassemi and Garzanti, 2019). In comparison to other deserts, the Karakum Desert is inhabited with animals and plants, mainly ephemeral plants, trees, and insects. Ninety-five percent of the area is more or less covered with vegetation, and the remaining 5% is sand dunes (Ghassemi and Garzanti, 2019). The annual average rainfall of Karakum Desert ranges from 70 to 150 mm. Its dry conditions are due to the fact that the coldest temperature of sand reaches −5°C, while the hottest temperature climbs from 55 to 88°C (Ghassemi and Garzanti, 2019). This dry but lively desert led many researchers to devise innovative projects that may benefit agriculture.

### Deserts in the Middle East, North Africa, and South America

There are 12 deserts located in the Middle East, North Africa, and South America: Anatolia Desert in Turkey, Saudi Arabia arid area, Beni Suef Governorate Desert, Eastern Desert and saline–alkaline desert in Egypt, Béni Abbès Desert, Béni Isguen Desert and Sahara Desert of Algeria, Sahara Desert in Libya, arid-saline sediment, and Sahara Desert in Tunisia (Supplementary Table 1). The Atacama Desert in Chile, being the primary source of new actinobacterial species, accounts for around 20% of all new species (23) and half of all novel compounds (25) in the past two decades. The Atacama Desert is a hyper-arid or an extremely hyper-arid area according to the ratio of mean annual rainfall to mean annual evaporation of less than 0.05 or even below 0.002 (Bull et al., 2016). Being the driest desert in the world, the Atacama Desert has high temperatures during the day that can easily reach over 40–50°C. The soil sample in the desert is very similar to the sample from Mars, with extremely low vegetation and organic carbon (about 2–50 μg of carbon per gram of soil) and high levels of UV radiation (Bull et al., 2016). Microbiologists are interested in the Atacama Desert as despite its harsh conditions, it harbors abundant and diverse actinobacteria with biotechnological potentials.

### Deserts in Other Regions

Other new actinobacteria were discovered from the Antarctic Desert (South Pole), arid Australian soils (Oceania), Baja California Desert in Mexico (North America), Namib Desert in Namibia (South Africa), and Sahara Desert in Chad (Central Africa) (Supplementary Table 1). The highest number of new species was recorded from the Sahara Desert in Chad (6) and arid Australian soils (5). The Sahara Desert is the world’s biggest hot desert, with extremely poor primary productivity, although it does sustain certain organisms that can adapt to arid environments, such as ephemerals (Schuster et al., 2006). The yearly precipitation in the Sahara Desert ranges from 0 to 7.62 cm, with some regions going for years without rain. The daily summer temperature of the Sahara Desert frequently exceeds 38°C during the day but drops to near freezing at night. Arid Australian soils are characterized mainly by mineral or skeletal soils almost without any organic matter (Morton et al., 2011). The hot and dry weather is typical for the Australian arid lands with low annual precipitation (380–635 mm), frequent heatwaves, and high levels of solar radiation.

## Diversity of Desert Actinobacteria From 2000 to 2021

**FIGURE 2:**
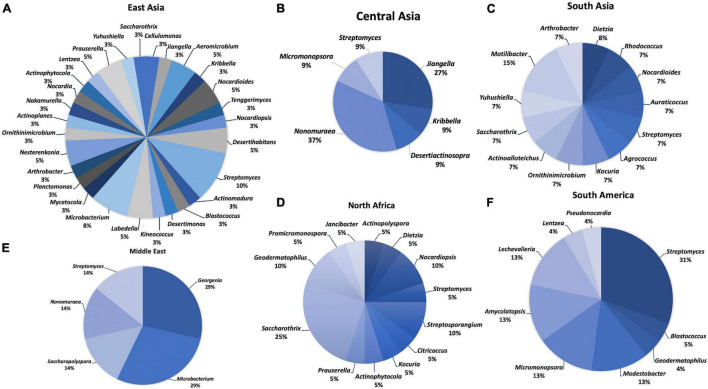
Distribution of desert actinobacteria from different geographical regions: **(A)** East Asia, **(B)** Central Asia, **(C)** South Asia, **(D)** North Africa, **(E)** Middle East, and **(F)** South America.

**FIGURE 3:**
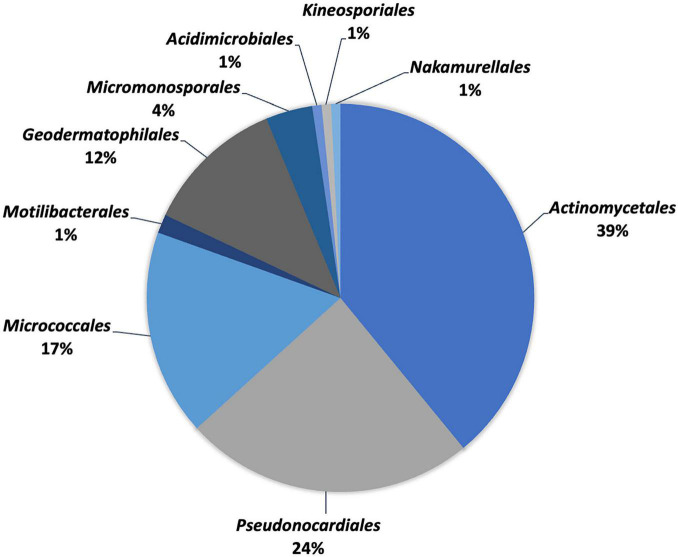
Percentage distribution of different orders of desert actinobacteria.

Extreme environmental conditions give rise to unique taxa (species and genera), where limited nutrient conditions significantly affect the structure of microbial communities (Hozzein and Goodfellow, 2007). From 2000 to 2021, 129 species in 9 orders, 24 families, and 52 genera were reported from deserts worldwide based on taxonomic studies (Table 1 and Figures 2, 3). Members of the order *Actinomycetales* (39%) represent the highest number of novel actinobacteria, followed by *Pseudonocardiales* (24%), *Micrococcales* (17%), *Geodermatophilales* (12%), *Micromonosporales* (4%), *Motilibacterales* (1%), *Acidimicrobiales* (1%), *Kineosporiales* (1%), and *Nakamurellales* (1%). Furthermore, members of the *Pseudonocardiaceae* family (23%) are the most abundant, followed by *Streptomycetaceae* (12%), *Geodermatophilaceae* (11%), *Microbacteriaceae* (7%), *Nocardioidaceae* (6%), *Streptosporangiaceae* (6%), *Micrococcaceae* (5%), *Micromonosporaceae* (4%), *Dietziaceae* (3%), *Jiangellaceae* (3%), *Nocardiopsaceae* (2%), *Propionibacteriaceae* (2%), *Bogoriellaceae* (2%), *Intrasporangiaceae* (2%), *Motilibacteraceae* (2%), *Thermomonosporaceae* (2%), *Actinopolysporaceae* (1%), *Cellulomonadaceae* (1%), *Ilumatobacteraceae* (1%), *Kineosporiaceae* (1%), *Nakamurellaceae* (1%), *Nocardiaceae* (1%), *Ornithinimicrobiaceae* (1%), and *Promicromonosporaceae* (1%).

**TABLE 1:** List of new actinobacteria isolated from desert environment from 2000 to 2021.

Order	Family	Genus	Species	References
*Acidimicrobiales*	*Ilumatobacteraceae*	*Desertimonas*	*Desertimonas flava*	Asem et al., 2018
*Actinomycetales*	*Actinopolysporaceae*	*Actinopolyspora*	*Actinopolyspora mzabensis*	Meklat et al., 2013
	*Cellulomonadaceae*	*Cellulomonas*	*Cellulomonas telluris*	Shi et al., 2020
	*Dietziaceae*	*Dietzia*	*Dietzia kunjamensis*	Mayilraj et al., 2006d
			*Dietzia lutea*	Li et al., 2009
		*Rhodococcus*	*Rhodococcus kroppenstedtii*	Mayilraj et al., 2006a
		*Williamsia*	*Williamsia* sp.	Guerrero et al., 2014
	*Jiangellaceae*	*Jiangella*	*Jiangella asiatica*	Saygin et al., 2020b
			*Jiangella aurantiaca*	
			*Jiangella ureilytica*	
			*Jiangella gansuensis*	Han et al., 2014
	*Nocardioidaceae*	*Aeromicrobium*	* *Aeromicrobium endophyticum*	Li et al., 2019a
			*Aeromicrobium halotolerans*	Yan et al., 2016
		*Kribbella*	*Kribbella deserti*	Sun et al., 2017
			*Kribbella turkmenica*	Saygin et al., 2019b
		*Nocardioides*	*Nocardioides deserti*	Tuo et al., 2015
			*Nocardioides thalensis*	Khan et al., 2017
			*Nocardioides vastitatis*	Liu et al., 2020b
		*Tenggerimyces*	*Tenggerimyces mesophilus*	Sun et al., 2015
	*Nocardiopsaceae*	*Nocardiopsis*	*Nocardiopsis alkaliphila*	Hozzein et al., 2004
			*Nocardiopsis benisuefensis*	Hozzein et al., 2014
			*Nocardiopsis sp.	Chen et al., 2015
	*Promicromonosporaceae*	*Promicromonospora*	*Promicromonospora panici*	Guesmi et al., 2021
	*Propionibacteriaceae*	*Auraticoccus*	*Auraticoccus cholistanensis*	Cheema et al., 2020
		*Desertihabitans*	*Desertihabitans aurantiacus*	Sun et al., 2019
			*Desertihabitans brevis*	Liu et al., 2020a
	*Streptomycetaceae*	*Streptomyces*	*Streptomyces aburaviensis*	Thumar et al., 2010
			*Streptomyces aridus*	Idris et al., 2017b
			*Streptomyces asenjonii*	Goodfellow et al., 2017
			*Streptomyces altiplanensis*	Cortés-Albayay et al., 2019
			*Streptomyces atacamensis*	Santhanam et al., 2012b
			*Streptomyces cahuitamycinicus*	Saygin et al., 2020c
			*Streptomyces desertarenae*	Li et al., 2019c
			*Streptomyces deserti*	Santhanam et al., 2012a
			*Streptomyces dengpaensis*	Li Y. et al., 2018
			*Streptomyces bullii*	Santhanam et al., 2013
			*Streptomyces fukangensis*	Zhang et al., 2013
			*Streptomyces fragilis*	Nithya et al., 2017
			*Streptomyces leeuwenhoekii*	Rateb et al., 2011a
			*Streptomyces netropsis	Abdelmoteleb and González-Mendoza, 2020
			*Streptomyces sannurensis*	Hozzein et al., 2011
			*Streptomyces taklimakanensis*	Yuan et al., 2020
	*Streptosporangiaceae*	*Desertiactinosopra*	*Desertiactinospora gelatinilytica*	Saygin et al., 2019a
		*Nonomuraea*	*Nonomuraea deserti*	Saygin et al., 2020d
			*Nonomuraea diastatica*	
			*Nonomuraea longispora*	
			*Nonomuraea mesophila*	
			*Nonomuraea terrae*	Ay, 2020
		*Streptosporangium*	*Streptosporangium algeriense*	Boubetra et al., 2016
			*Streptosporangium becharense*	Chaouch et al., 2016
	*Thermomonosporaceae*	*Actinomadura*	*Actinomadura deserti*	Cao et al., 2018
			*Actinomadura namibiensis*	Wink et al., 2003
*Geodermatophilales*	*Geodermatophilaceae*	*Blastococcus*	*Blastococcus atacamensis*	Castro et al., 2018a
			*Blastococcus deserti*	Yang et al., 2019
		*Geodermatophilus*	*Geodermatophilus africanus*	Montero-Calasanz et al., 2013b
			*Geodermatophilus arenarius*	Montero-Calasanz et al., 2012
			*Geodermatophilus chilensis*	Castro et al., 2018b
			*Geodermatophilus pulveris*	Hezbri et al., 2016
			*Geodermatophilus sabulis*	Hezbri et al., 2015
			*Geodermatophilus saharensis*	Montero-Calasanz et al., 2013c
			*Geodermatophilus siccatus*	Montero-Calasanz et al., 2013e
			*Geodermatophilus telluris*	Montero-Calasanz et al., 2013d
			*Geodermatophilus tzadienisis*	Montero-Calasanz et al., 2013a
		*Modestobacter*	*Modestobacter altitudinis*	Goliñska et al., 2020b
			*Modestobacter caceresii*	Busarakam et al., 2016b
			*Modestobacter excelsi*	Goliñska et al., 2020a
			*Modestobacter multiseptatus*	Mevs et al., 2000
*Kineosporiales*	*Kineosporiaceae*	*Kineococcus*	*Kineococcus xinjiangensis*	Liu et al., 2009
*Micrococcales*	*Bogoriellaceae*	*Georgenia*	*Georgenia deserti*	Hozzein et al., 2018
			*Georgenia alba*	Li et al., 2019d
	*Intrasporangiaceae*	*Janibacter*	*Janibacter* sp.	Khessairi et al., 2014
		*Ornithinicoccus*	*Ornithinicoccus halotolerans*	Zhang et al., 2016
	*Microbacteriaceae*	*Agrococcus*	*Agrococcus lahaulensis*	Mayilraj et al., 2006e
		*Labedella*	* *Labedella phragmitis*	Li et al., 2019b
			* *Labedella populi*	
		*Microbacterium*	*Microbacterium album*	Yang et al., 2018b
			*Microbacterium deserti*	
			*Microbacterium halophytorum	Li Y. R. et al., 2018
			*Microbacterium suaedae*	Zhu et al., 2019
			*Microbacterium karelineae*	Zhu et al., 2021
		*Mycetocola*	*Mycetocola manganoxydans*	Luo et al., 2012
		*Planctomonas*	*Planctomonas deserti*	Liu et al., 2019b
	*Micrococcaceae*	*Arthrobacter*	*Arthrobacter deserti*	Hu et al., 2016
			*Arthrobacter mobilis*	Ye et al., 2020
		*Citricoccus*	*Citricoccus alkalitolerans*	Li et al., 2005
		*Kocuria*	*Kocuria aegyptia*	Li et al., 2006
			*Kocuria himachalensis*	Mayilraj et al., 2006b
		*Nesterenkonia*	*Nesterenkonia populi*	Liu et al., 2015b
			*Nesterenkonia rhizosphaerae*	Wang et al., 2014
	*Ornithinimicrobiaceae*	*Ornithinimicrobium*	*Ornithinimicrobium kibberense*	Mayilraj et al., 2006c
*Micromonosporales*	*Micromonosporaceae*	*Actinoplanes*	*Actinoplanes deserti*	Habib et al., 2018
		*Micromonospora*	*Micromonospora acroterricola*	Carro et al., 2019b
			*Micromonospora arida*	Carro et al., 2019a
			*Micromonospora inaquosa*	
			*Micromonospora deserti*	Saygin et al., 2020a
*Motilibacterales*	*Motilibacteraceae*	*Motilibacter*	*Motilibacter aurantiacus*	Liu et al., 2020c
			*Motilibacter deserti*	
*Nakamurellales*	*Nakamurellaceae*	*Nakamurella*	*Nakamurella deserti*	Liu et al., 2019a
*Pseudonocardiales*	*Nocardiaceae*	*Nocardia*	*Nocardia sp.*	Zhang et al., 2020
	*Pseudonocardiaceae*	*Actinoalloteichus*	*Actinoalloteichus spitiensis*	Singla et al., 2005
		*Actinophytocola*	*Actinophytocola algeriensis*	Bouznada et al., 2016b
			*Actinophytocola gilvus*	Sun et al., 2014
		*Amycolatopsis*	*Amycolatopsis australiensis*	Tan et al., 2006
			*Amycolatopsis deserti*	Busarakam et al., 2016a
			*Amycolatopsis granulosa*	Zucchi et al., 2012a
			*Amycolatopsis ruanii*	
			*Amycolatopsis thermalba*	
			*Amycolatopsis thermophila*	Zucchi et al., 2012b
			*Amycolatopsis viridis*	
			*Amycolatopsis vastitatis*	Idris et al., 2018
		*Lechevalieria*	*Lechevalieria atacamensis*	Okoro et al., 2010
			*Lechevalieria deserti*	
			*Lechevalieria roselyniae*	
		*Lentzea*	*Lentzea isolaginshaensis*	Wang et al., 2019
			*Lentzea chajnantorensis*	Idris et al., 2017c
		*Prauserella*	*Prauserella endophytica*	Liu et al., 2015a
			*Prauserella isguenensis*	Saker et al., 2015
			*Prauserella shujinwangii*	Liu et al., 2014
		*Pseudonocardia*	*Pseudonocardia nigra*	Trujillo et al., 2017
		*Saccharopolyspora*	*Saccharopolyspora deserti*	Yang et al., 2018a
		*Saccharothrix*	*Saccharothrix algeriensis	Zitouni et al., 2004
			*Saccharothrix deserti*	Liu et al., 2020
			*Saccharothrix ghardaiensis*	Bouznada et al., 2017
			*Saccharothrix hoggarensis*	Boubetra et al., 2013b
			*Saccharothrix isguenensis*	Bouznada et al., 2016a
			*Saccharothrix tamanrassetensis*	Boubetra et al., 2015
			*Saccharothrix tharensis*	Ibeyaima et al., 2018
		*Yuhushiella*	*Yuhushiella deserti*	Mao et al., 2011
			*Yuhushiella* sp.	Ibeyaima et al., 2016

*Species isolated from plants in desert area.

The two most dominant actinobacteria recovered from deserts are *Streptomyces* and *Geodermatophilus*. *Streptomyces*, the largest genus of actinobacteria, has the greatest number (16) of validly described desert actinobacterial species. To survive in desert environments, members of the genus *Streptomyces* have evolved a variety of tolerance mechanisms and bioactive compound potential. Currently, all reported bioactive compounds were derived from desert streptomycetes (Bérdy, 2005). For example, *Streptomyces aburaviensis* (Thumar et al., 2010), *Streptomyces fukangensis* (Zhang et al., 2013), and *Streptomyces sannurensis* (Hozzein et al., 2011) are alkaliphilic actinomycetes, with optimum growth at pH 9 to 11.5. *S*. *aburaviensis* (Thumar et al., 2010) and *Streptomyces desertarenae* (Li et al., 2019c) are halotolerant species that can adapt to living in high-saline environments (7–15% w/v NaCl), while *Streptomyces asenjonii* (Abdelkader et al., 2018) and *Streptomyces leeuwenhoekii* (Rateb et al., 2011a) were gifted for the synthesis of bioactive compounds with novel chemical structures. It should be noted that the majority of streptomycetes (7) was discovered from the Atacama Desert, including *Streptomyces altiplanensis* (Cortés-Albayay et al., 2019), *Streptomyces atacamensis* (Santhanam et al., 2012b), *Streptomyces deserti* (Santhanam et al., 2012a), and *Streptomyces bullii* (Santhanam et al., 2013), which adapted to the alkaline environments with optimum growth in pH of up to 11; *Streptomyces aridus* (Idris et al., 2017b) is capable of growth in a temperature range between 10 and 40°C, and *S. asenjonii* (Abdelkader et al., 2018) and *S. leeuwenhoekii* (Gran-Scheuch et al., 2018) produced antibacterial metabolites under desert growth conditions. Another dominant group of desert actinobacteria is the genus *Geodermatophilus* with nine new species identified within the same period. Members of this taxon have demonstrated radiation-resistant capabilities, such as *Geodermatophilus pulveris* BMG 825^T^ and *Geodermatophilus sabuli* BMG 8133^T^ which are both gamma radiation resistant, whereas *Geodermatophilus tzadiensis* CF5/2^T^ is a UV-radiation-resistant actinobacterium (Hezbri et al., 2015; Hezbri et al., 2016).

### Actinobacterial Diversity in East Asia

Most new actinobacterial species (39) were discovered from East Asia, and they were classified into 28 genera (Figure 2). The highest number of novel species was isolated from the Taklamakan Desert (14), belonging to 13 genera: *Aeromicrobium*, *Desertihabitans*, *Labedell*, *Microbacterium*, *Mycetocola*, *Nakamurella*, *Nesterenkonia*, *Nocardioides*, *Nocardioides*, *Nocardiopsis*, *Planctomonas*, *Prauserella*, and *Streptomyces*. Among them, two new species, *Labedella phragmitis* and *Labedella populi*, were obtained from the surface-sterilized flowers of *Phragmites australis* (reed) and branches of *Populus euphratica*, respectively (Li et al., 2019b). Surface-sterilized leaves of *P. australis* (reed) were the isolation source of an endophytic actinobacterium, *Aeromicrobium endophyticum* (Li et al., 2019a). In addition, two actinobacteria, *N. deserti* 12Sc4-1^T^ and *N. deserti* SC8A-24^T^ were collected from the rhizosphere of *Reaumuria* and *Alhagi sparsifolia*, respectively. The latter strain showed desiccation tolerance properties (Tuo et al., 2015; Liu et al., 2019a). NaCl tolerance is a common feature among desert actinobacteria; for example, *Desertihabitans brevis* 16Sb5-5^T^, isolated from a sand sample in the Taklamakan Desert, tolerates up to 10% NaCl (Liu et al., 2020a). Members of the following genera, *Actinomadura*, *Kineococcus*, *Nocardia*, *Ornithinicoccus*, *Prauserella*, *Saccharothrix*, and *Yuhushiella*, were isolated from Xinjiang Desert. Halotolerant actinobacteria are able to grow under salinity stress, for example, *Ornithinicoccus halotolerans* EGI 80423^T^ from Xinjiang Desert which grew in up to 14% NaCl (Zhang et al., 2016). Four genera, *Blastococcus*, *Desertimonas*, *Microbacterium*, and *Streptomyces*, were isolated from the Gurbantunggut Desert. *Actinoplanes*, *Aeromicrobium*, and *Arthrobacter* were recovered from the Turpan Desert. One new species, *Aeromicrobium halotolerans* YIM Y47^T^ is able to grow in 0%–7% NaCl (Yan et al., 2016). Members of the genera *Cellulomonas* and *Desertihabitans* were isolated from the Badain Jaran Desert, while *Nesterenkonia* and *Streptomyces* strains were reported from the saline–alkaline desert of Fukang. One new species, *Desertihabitans aurantiacus* CPCC 204711^T^ from the sand of Badain Jaran Desert, could tolerate up to 5% NaCl (Sun et al., 2019). An alkaliphilic actinobacterium, *Nesterenkonia rhizosphaerae* YIM 80379^T^, was reported from the rhizosphere soil of *Reaumuria soongorica* grown in the saline–alkaline desert of Fukang (Wang et al., 2014). This strain could grow in up to 15% NaCl and pH 9–10. Another alkaliphile, *S. fukangensis* EGI 80050^T^, was also isolated from Fukang Desert with optimal growth observed at pH 9.0–10.0 and 2.5–5.0% NaCl (Zhang et al., 2013). Members of the genera *Jiangella*, *Kribbella*, *Lentzea*, and *Streptomyces* were reported from Gansu Desert, Hangjin Banner Desert, Isolaginsha Desert, and Tibet Autonomous Color Desert, respectively. In Hangjin Banner Desert, a strictly aerobic and non-motile actinobacterium, *Kribbella deserti* SL15-1^T^, was isolated from the rhizosphere of *Ammopiptanthus mongolicus* (Sun et al., 2017).

### Actinobacterial Diversity in Central and South Asia

Eleven new species of the genera *Jiangella*, *Kribbella*, *Desertiactinospora*, *Nonomuraea*, *Micromonospora*, and *Streptomyces* have been discovered from the Karakum Desert in Central Asia. In South Asia, members of the genera *Actinoalloteichus*, *Agrococcus*, *Kocuria*, *Ornithinimicrobium*, and *Rhodococcus* were found from the Lahaul–Spiti Valley cold desert of the Indian Himalayas. Members of the genera *Auraticoccus*, *Arthrobacter*, and *Motilibacter* were recovered from the Cholistan Desert in Pakistan, while *Saccharothrix* and *Yuhushiella* were discovered from the Indian Thar Desert. Novel species belonging to the genera *Dietzia*, *Nocardioides*, and *Streptomyces* were isolated from the Kunjam Pass cold desert of the Indian Himalayas, the Thal Desert in Pakistan, and the Kutch saline Desert in India, respectively. *S. aburaviensis* strain Kut-8 isolated from the saline desert of Kutch is a halotolerant and alkaliphilic actinobacterium that could grow under saline (15% NaCl) and alkaline (pH 9) conditions (Thumar et al., 2010).

### Actinobacterial Diversity in the Middle East, North Africa, and South America

In the Middle East, *Nonomuraea terrae* was isolated from the Anatolia Desert, Turkey, and six new species belong to four genera: *Georgenia*, *Microbacterium*, *Saccharopolyspora*, and *Streptomyces*, which were discovered from the Saudi Arabia arid desert. Three new species, *Georgenia alba* SYSU D8008^T^ (grew in up to 7% NaCl) (Li et al., 2019d), *Georgenia deserti* SYSU D8004^T^ (grew in up to 17% NaCl) (Hozzein et al., 2018), and *Saccharopolyspora deserti* SYSU D8010^T^ (grew in up to 22% NaCl) (Liu et al., 2020), are reported as halotolerant actinobacteria. Representative members of 12 genera were obtained from nine deserts in North Africa. Members of the genera *Actinophytocola*, *Saccharothrix*, and *Streptosporangium* were isolated from the Sahara Desert in Algeria, whereas *Citricoccus*, *Dietzia*, *Nocardiopsis*, and *Streptomyces* were reported from the Eastern Desert of Egypt. A member of the genus *Actinopolyspora, Actinopolyspora mzabensis* H55^T^ isolated from an Algerian Saharan soil, could tolerate up to 32% NaCl (Saygin et al., 2019a). Alkaliphiles include *Citricoccus alkalitolerans* YIM 70010^T^ (pH 8–9) (Li et al., 2005), *Nocardiopsis alkaliphila* YIM 80379^T^ (pH 9.5–10) (Hozzein et al., 2004), and *S. sannurensis* WS 51^T^ (pH 9.5–10) (Hozzein et al., 2011), which were isolated from the Eastern Desert of Egypt. *Saccharothrix algeriensis* was cultured from a soil sample of palm groves in the Saharan Desert (Zitouni et al., 2004). *Geodermatophilus* and *Promicromonospora* strains were recovered from the Sahara Desert in Tunisia. Other genera, namely, *Nocardiopsis*, *Janibacter*, *Kocuria*, *Prauserella*, and *Streptosporangium*, were described from the Beni Suef Governorate Desert in Egypt, the arid-saline sediment in Tunisia, the saline–alkaline desert in Egypt, the Béni Isguen Desert, and the Béni Abbès Desert in Algeria, respectively. The alkaliphilic strain of *Nocardiopsis benisuefensis* WS65 was isolated from the Beni Suef Governorate Desert in Egypt (Hozzein et al., 2014), while a halotolerant actinobacterium *Prauserella isguenensis* H225^T^ (grew in up to 25% NaCl) was recovered from the Béni Isguen (Mzab) desert in Algeria (Saker et al., 2015). Twenty-three new species belonging to nine genera (*Amycolatopsis*, *Blastococcus*, *Geodermatophilus*, *Lechevalieria*, *Lentzea*, *Micromonospora*, *Modestobacter*, *Pseudonocardia*, and *Streptomyces*) were reported and described from the Atacama Desert in Chile. One new species, *Pseudonocardia nigra* ATK03^T^, was isolated from the rock surface in the Atacama Desert (Trujillo et al., 2017). Two new species recognized as thermophiles, namely, *Amycolatopsis ruanii* NMG112^T^ and *Amycolatopsis thermalba* SF45^T^, could survive under high temperatures (Zucchi et al., 2012a). Another new species, *Modestobacter caceserii* KNN 45-2b^T^ from the Atacama Desert in Chile, highly adapted to high osmotic stress, cold shock, heat and desiccation, low nutrient, low organic carbon, and high levels of UV radiation (Busarakam et al., 2016b).

### Actinobacterial Diversity in Other Regions

*Actinomadura namibiensis* was isolated from the Namibian Namib Desert in South Africa. *Streptomyces netropsis* was first discovered in North America in the Baja California Desert of Mexico. The Antarctic Desert at the South Pole yielded one new species of *Modestobacter multiseptatus* and *Williamsia* sp., where the former strain was a budding-like psychrophilic actinobacterium isolated from the rock of the Transantarctic Mountains (Mevs et al., 2000). *Williamsia* sp. D3 was considered as a psychrotolerant, able to grow under at a low temperature of 15°C (Guerrero et al., 2014). All new species from the Sahara Desert of Chad in Central Africa and the arid Australian Desert in Oceania were classified in the genera *Geodermatophilus* and *Amycolatopsis*, respectively. Six new species belonging to the *Geodermatophilus* genus are *Geodermatophilus africanus* (Montero-Calasanz et al., 2013b), *Geodermatophilus arenarius* (Montero-Calasanz et al., 2012), *Geodermatophilus saharensis* (Montero-Calasanz et al., 2013c), *Geodermatophilus siccatus* (Montero-Calasanz et al., 2013e), *Geodermatophilus telluris* (Montero-Calasanz et al., 2013d), and *G. tzadiensis* (Montero-Calasanz et al., 2013a). Two *Geodermatophilus* species, *G*. *arenarius* CF5/4^T^ (Montero-Calasanz et al., 2012) and *G. siccatus* CF6/1^T^ (Montero-Calasanz et al., 2013e) from the Sahara Desert in Chad, have high drought tolerance ability. Members of the genus *Amycolatopsis* were described as *Amycolatopsis australiensis* (Tan et al., 2006), *A. deserti* (Busarakam et al., 2016a), *Amycolatopsis granulosa* (Zucchi et al., 2012a), *Amycolatopsis thermophila*, and *Amycolatopsis viridis* (Zucchi et al., 2012b). Most of them were recognized as thermophiles that could survive under high temperature, including *A. deserti* GY024^T^, *A. granulosa* GY307^T^, *A. thermophila* GY088^T^, and *A. viridis* GY115^T^, as well as *A. ruanii* NMG112^T^ and *A. thermalba* SF45^T^ from the Atacama Desert in Chile.

## Isolation and Taxonomic Characterization of Desert Actinobacteria

**FIGURE 4:**
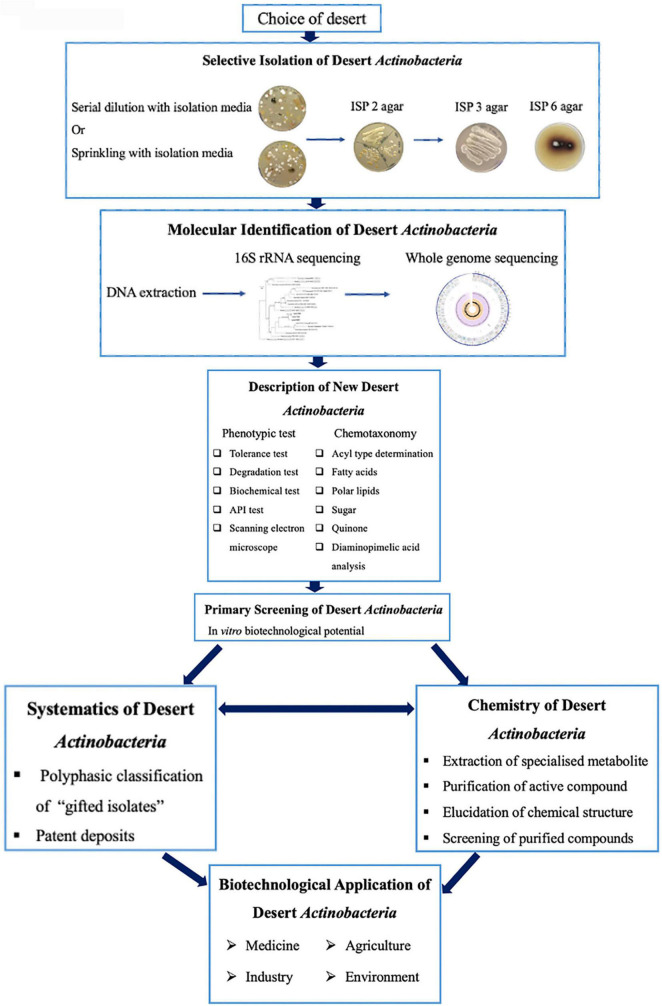
Bioprospecting strategy for desert actinobacteria (modified from Goodfellow and Fiedler, 2010).

A bioprospecting strategy to recover actinobacteria from the desert is summarized in Figure 4. Most procedures involve the selection of samples from the desert, isolation on selective media, and incubation and selection of representative colonies from the isolation plates for further study (Goodfellow, 2010; Goodfellow and Fiedler, 2010; Tiwari and Gupta, 2013). The low occurrence of rare actinobacteria on the isolation plates clearly reflected the limitations of isolation, cultivation, and maintenance using conventional approaches, which fail to provide optimal growth conditions for desert actinobacteria. Thus, a better or improved isolation method is significant for increasing the number of diverse actinobacterial taxa, which remains an effective strategy for natural product discovery (Jensen, 2010; Jensen et al., 2015).

### Selective Isolation of Desert Actinobacteria

#### Serial Dilution vs. Sprinkling Technique

In general, selective isolation of actinobacteria from desert samples followed the procedure described by Okoro et al. (2009). Serial dilution is commonly used to prepare desert samples for inoculation. Aliquots of soil suspension prepared from serial dilutions were plated onto the selective isolation media, pre-dried in a laminar flow hood for 15 min (Vickers and Williams, 1987). However, there are certain errors associated with sample preparation using a serial dilution method (Ben-David and Davidson, 2014). The first error is related to the initial sample size. If the sample size is larger or smaller than the nominal diluent, under-dilution or over-dilution errors will occur. Similarly, diluent types, mixing method, and mixing time would also cause the same problems. Other errors may occur due to the pipetting technique. It is possible that a non-representative sample will be transferred to the new dilution and finally inoculated on the isolation plates. These errors would lead to a low number of colonies on the isolation plates. Recently, serial dilution, which is part of the conventional culture procedure (CCP), was clearly demonstrated to have limited selectively for even the isolation of members of the genus *Streptomyces*, the most dominant desert actinobacteria (Li et al., 2021).

The sprinkling technique is proposed here as an improved isolation method since few reports have found that it is a simpler and less error-prone way to obtain desert actinobacteria compared to the traditional serial dilution technique, especially for the desert taxa, which may prefer to develop in a reduced-moisture environment, similar to that of the desert, rather than a suspension in a diluent. For example, a greater number of actinobacteria were recorded from selective isolation media using the sprinkling inoculation technique as opposed to the suspensions of the desert sample from Atacama Desert as summarized in Table 2 (Xie, 2017). This comparative study was carried out using the same selective media supplemented with the same antibiotics under the same incubation conditions. The number of actinobacteria colonies obtained from sprinkling was 10–20 times higher than that acquired via serial dilution. Higher relative abundance was also observed from the sprinkling technique with actinobacterial count representing 85–100% of total bacteria on the isolation plates. The obtained actinobacteria showed antimicrobial activity that is effective against a panel of bacteria, namely, *Bacillus subtilis*, *Escherichia coli*, *Pseudomonas fluorescens*, *Saccharomyces cerevisiae*, and *Staphylococcus aureus*. It is evident that the sprinkling approach clearly provides a higher number of bioactive actinobacteria (141) compared to the serial dilution (97). Again, the number of endophytic actinobacteria acquired from the sprinkling technique is approximately 1.1 times higher than the total population obtained from two improved serial dilution procedures (Qin et al., 2009). Indeed, the isolation medium directly seeded with mineral particles from Atacama Desert soils was found to obtain a higher actinobacterial count than did the serial dilution plates (Idris, 2016). This sprinkling technique allows actinobacterial cells to attach to soil particles in direct contact with the culture media, which increases the isolation efficiency by promoting colony development on the isolation plates (Duddington, 1955; Bailey and Gray, 1989; Qin et al., 2009).

**TABLE 2:** Comparison of serial dilution and sprinkling technique with number and relative abundance of actinobacterial colonies (cfus per gram of dry weight environmental samples) growing on selective media prior to incubation at 28°C for 14 days (modified from Xie, 2017).

Selective medium	Serial dilution			Sprinkling		
	Number of actinobacteria	Relative abundance (%, compared to the total bacteria)	Number of bioactive actinobacteria	Number of actinobacteria	Relative abundance (%, compared to the total bacteria)	Number of bioactive actinobacteria
**Valle de la Luna composite sample (VL1 + VL2 + VL3)**						
Gauze’s no. 1 agar + nalidixic acid (10 μg/ml)	12	65	**5**	**77**	**85**	2
Starch casein agar	31	**72**	3	**87**	62	**6**
HV agar	5	50	0	**80**	**86**	**10**
**Valle de la Luna rock scrappings (VLR)**						
Gauze’s no. 1 agar + nalidixic acid (10 μg/ml)	11	57	3	**177**	**100**	**6**
Starch casein agar	31	45	8	**190**	**76**	**21**
HV agar	1	29	0	**123**	**95**	**14**
**Paranal/Paposo composite sample (POP1 + POP2)**						
Gauze’s no. 1 agar + nalidixic acid (10 μg/ml)	6	75	0	**53**	**76**	**8**
Starch casein agar	39	41	1	**393**	**60**	**9**
HV agar	5	5	0	**107**	**89**	**7**
**Yungay composite sample (Y6, 2 and 30 cm)**						
Gauze’s no. 1 agar + nalidixic acid (10 μg/ml)	196	83	**35**	**513**	**86**	27
Starch casein agar	72	50	18	**453**	**59**	**25**
HV agar	63	43	**24**	**310**	**85**	12

*The bolded number indicates the highest value in the comparison of serial dilution and sprinkling technique under the same column items. All media were supplemented with cycloheximide and nystatin (each at 25 μg/ml).*

#### Selective Isolation Media

To maximize the number of actinobacteria, a variety of culture media were employed for the isolation of desert actinobacteria. The effectiveness of the isolation media is closely related to the ecological characteristics of novel actinobacteria from the desert. The media must meet the growth requirements of the target taxa; for example, halophilic strains prefer growth in high-salt-containing media. Selective isolation media resembling the characteristics of the study site increase the success rate of isolation. Carbon and nitrogen sources, growth factors, mineral salts, vitamins, and water are all essential components for the growth of desert actinobacteria on any culture medium (Bonnet et al., 2019). In this aspect, media can be grouped into nutrient-rich media and nutrient-poor media. It is worthy to note that the most effective selective isolation media are specifically designed based on the needs and preferences of actinobacteria, notably nutrient requirements and tolerance preferences of the target taxa. Conventional nutrient-rich media have been widely used to cultivate actinobacteria with limited success. Glucose yeast extract (GYE) agar is recommended as the most suitable medium that can result in a high ratio of actinobacterial count (Agate and Bhat, 1963). Starch casein agar is preferable, especially for saccharolytic organisms, since it contains starch as the sole carbon source as well as sufficient minerals for protein production (Lee et al., 2014). However, recent studies showed that the normally slow-growing rare genera of *Actinobacteria* and *Proteobacteria* failed to grow on commonly used rich media, but growth was observed only from low-nutrient media (Asem et al., 2018; Salam et al., 2021). Approximately 100 novel actinobacteria have been isolated from nutrient-poor media (Salam et al., 2021). This information strongly suggested that nutrient-poor media are more effective for the growth of desert strains as compared to those previously used nutrient-rich media (Bérdy, 2005; Bredholdt et al., 2007; Hozzein et al., 2008). Nutrient-poor media such as soil extract media and a new minimal medium (MM), which reflect the extreme environmental conditions in the desert with low organic matter content, were superior to currently used isolation media for desert actinobacteria (Hozzein et al., 2008). Similarly, humic acid–vitamin (HV) media are also considered as low-nutrient media and are more effective for the isolation of sporulating actinobacteria than are high-nutrient media (Hayakawa, 2008).

Approximately 40 isolation media have been utilized over the last two decades to isolate diverse actinobacterial taxa from the desert (Supplementary Table 3). Reasoner’s 2A (R2A) agar was the most often used media for desert actinobacterial isolation, representing 25% of reported genera (13): *Blastococcus* (Yang et al., 2019), *Geodermatophilus* (Montero-Calasanz et al., 2013b), *Georgenia* (Li et al., 2019d), *Jiangella* (Saygin et al., 2020b), *Microbacterium* (Yang et al., 2018b), *Micromonospora* (Carro et al., 2019b), *Nocardioides* (Liu et al., 2020), *Nonomuraea* (Saygin et al., 2020c), *Ornithinicoccus* (Zhang et al., 2016), *Saccharopolyspora* (Yang et al., 2018a), *Streptomyces* (Li et al., 2019c), *Tenggerimyces* (Sun et al., 2015), and *Williamsia* (Guerrero et al., 2014). Other media often employed for the isolation of desert taxa include humic acid–vitamin agar and chitin–vitamin agar, which are low-nutrient media comparable to desert environments. Sometimes, media were specially formulated to adjust the nutrient contents to meet the requirements of desert actinobacteria, such as chitin medium, 0.1× tryptic soy agar (TSA) medium, tenfold-diluted trypticase soy broth (TSB) agar, modified 0.3× marine broth agar, and 1/5-strength R2A agar, which were successfully used to recover members of the genera *Labedella* (Li et al., 2019b), *Desertimonas* (Asem et al., 2018), *Kineococcus* (Liu et al., 2009), *Mycetocola* (Luo et al., 2012), and *Tenggerimyces* (Sun et al., 2015). It is worth noting that SM1, SM2, and SM3 agar were specifically designed for the isolation of the *Amycolatopsis* genus (Tan et al., 2006; Zucchi et al., 2012a), whereas medium A was used to recover the alkaliphilic and alkaline-resistant actinobacteria, such as *C. alkalitolerans* (Li et al., 2005), *Nocardiopsis alkaliphile* (Hozzein et al., 2004), and *S. sannurensis* (Hozzein et al., 2011). In addition, media such as starch casein agar and raffinose-histidine agar were used to promote the growth of desert taxa by employing casein and raffinose (selective macromolecules) as nitrogen and carbon sources, respectively. *Saccharothrix tharensis* (Thumar et al., 2010) and *Yuhushiella* sp. (Ibeyaima et al., 2016) were recovered from starch casein agar, while *S. atacamensis* (Santhanam et al., 2012b) and *Streptomyces deserti* (Santhanam et al., 2012a) were isolated from raffinose-histidine agar. Recently, a highly selective culture strategy for the isolation of *Streptomyces* spp. from desert samples was proposed based on the use of minimal medium, actinobacteria isolation agar, and starch casein agar in combination with a cocktail of selective inhibitors (Li et al., 2021). These authors claimed to increase almost fourfold the number and phylotypes of *Streptomyces* strains isolated from the Gurbantunggut Desert as compared to the conventional approach.

#### Selective Antibiotics

Another method for increasing the effectiveness of selective isolation of desert actinobacteria is to supplement appropriate antibiotics in the media to enhance the recovery of actinobacteria. Gram-positive (*Bacillus* spp.) and Gram-negative bacteria (*Enterobacteria* and *Escherichia*), as well as fungi (*Alternaria*, *Aspergillus*, *Cladosporium*, *Fusarium*, and *Penicillium*) and yeast, are all frequent contaminants (Bonnet et al., 2019). Thus, selective antibiotics are used to prevent the development of these unwanted fast-growing microorganisms (both bacteria and fungi) that may outcompete actinobacteria on the isolation plates (Entis, 2002; Bonnet et al., 2019). Various antibiotics have been employed to prevent the contaminants from reducing the growth of the desired actinobacteria, summarized in Supplementary Table 3. Williams and Davies (1965) initially recommended that a mixture of nystatin (50 μg/ml) and actidione (cycloheximide, 50 μg/ml) was suitable for the enumeration of soil actinobacteria, along with polymyxin B-sulfate (50 μg/ml) and sodium penicillin (10 μg/ml). After that, amphotericin B (20 μg/ml), actidione, calcium propionate (30 mg/ml), cycloheximide (25 μg/ml), nystatin (25 μg/ml), and potassium dichromate (45 mg/L) have been commonly used as antifungal and yeast agents. Penicillin G, bacitracin, and vancomycin were used against Gram-positive bacteria (Bonnet et al., 2019). Polymyxin B (25 mg/L), colistin (2 μg/ml), nalidixic acid (25 mg/l), and ceftazidime (8 μg/ml) were effective for growth inhibition of Gram-negative bacteria. Several antibiotics are active against both Gram-positive and Gram-negative bacteria, including cefalotin, cefamandole, cefixime, ticarcillin, trimethoprim, nitrofurantoin, chloramphenicol, rifampicin (5 μg/ml), oxytetracycline, erythromycin, neomycin (4 μg/ml), gentamicin, fosfomycin, and novobiocin (25 μg/ml) (Bonnet et al., 2019). For the isolation of members of the *Amycolatopsis* genus, SM1 agar was designed to be supplemented with neomycin (1 μg/ml) and cycloheximide and nystatin (each at 25 μg/ml); SM2 agar with neomycin (4 μg/ml), D (+) melezitose (1%, w/v), and nystatin (50 μg/ml); and SM3 agar with cycloheximide (50 μg/ml), nalidixic acid (10 μg/ml), novobiocin (10 μg/ml), and nystatin (50 μg/ml) (Tan et al., 2006). Cycloheximide (50 μg/ml), nalidixic acid (50 μg/ml), and nystatin (100 μg/ml) are often used and responsible for the isolation of 50% of the reported desert actinobacterial genera (27) as shown in Supplementary Table 3. Recently, a combination of cycloheximide (25 mg/l), nalidixic acid (25 mg/l), and potassium dichromate (25 mg/L) was used as part of a highly selective culture strategy for the isolation of *Streptomyces* spp. from Gurbantunggut Desert (Li et al., 2021).

#### Incubation Conditions

Incubation conditions, in particular temperature and incubation duration, are the other important factors in the improvement of isolation (Fang et al., 2017; Ríos-Castillo et al., 2020; Supplementary Table 3). It is obvious that the optimal temperature for most actinobacteria is 25–30°C, which could be used as a general incubation temperature for desert actinobacteria. However, extremophilic actinobacteria with specific preference for growth temperature do exist in the desert biomes. For example, one psychrophile, *M. multiseptatus* (Mevs et al., 2000), prefers to grow at a lower temperature (19–21°C), while a thermophile, *Amycolatopsis vastitatis* (Busarakam et al., 2016a), requires an incubation temperature of 45°C. Fourteen new species were recovered from the incubation temperature of 10–50°C. The highest number of new actinobacterial species was obtained at 25–30°C, which is the optimum growth temperature for the majority of desert actinobacteria. Another key factor for selective isolation is the incubation duration. The incubation time which allows actinobacteria to fully grow on the isolation plates ranged from 3 days to 3 months (Supplementary Table 3). The fast-growing species are generally allowed abundant growth after 3–14 days, such as *Auraticoccus cholistanensis* (Cheema et al., 2020), *Blastococcus deserti* (Yang et al., 2019), *C. alkalitolerans* (Li et al., 2005), *G. deserti* (Hozzein et al., 2018), *Janibacter* sp. (Khessairi et al., 2014), *Kineococcus xinjiangensis* (Liu et al., 2009), and *K. deserti* (Sun et al., 2017). Slow-growing genera such as *Labedella* (Li et al., 2019b), *Motilibacter* (Liu et al., 2020c), *Modestobacter* (Mevs et al., 2000), *Nonomuraea* (Saygin et al., 2020c), and *Williamsia* (Guerrero et al., 2014) may need more than 4 weeks to fully develop. Nevertheless, the majority of actinobacterial taxa grow well between 2 and 4 weeks. Thus, a specific incubation time allows the recovery of different groups of desert actinobacteria with varied growth rates.

### Taxonomic Characterization of Desert Actinobacteria

In general, taxonomic characterization of desert actinobacteria is started with the acquisition of a pure culture of each isolate. Putative actinobacterial colonies from the isolation plates are purified through several rounds of transfer to suitable culture media. Pure isolates of actinobacteria usually develop with different morphological features after being incubated for 2–4 weeks. Morphological characterization of these pure isolated strains was generally observed according to the guidelines proposed by the International *Streptomyces* Project (ISP), including aerial spore mass, substrate mycelial and diffusible pigments, growth, and formation of melanin pigments (Shirling and Gottlieb, 1966). All isolates were further assigned to single- and multi-membered color groups based on the color of the aerial spore mass and substrate mycelial and diffusible pigments. Representative isolates from each morphotype of these assigned color groups are selected for comparative 16S rRNA gene sequence analysis.

The 16S rRNA gene sequencing analysis is routinely the first step for the identification of desert actinobacteria. Genomic DNA of representative isolates can be extracted by various methods such as the phenol-chloroform extraction (Sagova-Mareckova et al., 2008) or solid-phase DNA extraction method such as the bead beating technique (Fujimoto et al., 2004). Several DNA extraction kits are commercially available. The commonly used kits include the MN DNA extraction kit (Macherey-Nagel, Düren, Germany) (Nithya et al., 2018) and Qiagen Genomic 500 DNA kit (Qiagen, Hilden, Germany) (Jiao et al., 2017). Nucleotide primers for 16S rRNA gene amplification were summarized in Table 2 including primers 27F and 1492R, which are commonly employed in the study of actinobacteria from deserts.

The information from the 16S rRNA gene sequence is also valuable in delineating actinobacteria from the subspecies to genus levels (Hayashi Sant’Anna et al., 2019). Strains showing less than 98.7% similarity are generally assigned as potential new species (Chun et al., 2018). However, the 16S rRNA genes of members of some closely related species can be too conserved that they cannot be used to differentiate between strains at the species level. Strains of related species with almost identical 16S rRNA gene sequences might belong to different genomic species, as exemplified by members of the genera *Amycolatopsis* (Sangal et al., 2018) and *Streptomyces* (Idris et al., 2017b). It is recommended to obtain a genome sequence for comparison to circumscribe novel species. Nevertheless, 16S rDNA sequence data remain invaluable as they can be used to select appropriate reference strains for whole-genome analysis, thereby reducing the number of marker strains that need to be examined.

The description of new taxa is based on application of the polyphasic taxonomic approach based on a combination of genotypic and phenotypic data. Genotypic data are derived from analyses of nucleic acids and phenotypic data from cultural, chemotaxonomic, morphological, nutritional, and other expressed features. Phenotypic tests involve several biochemical properties and ranges of physiological properties such as pH, temperature, and the concentration of NaCl. Enzymatic activities are currently investigated using the API ZYM (bioMérieux) commercial system (Saygin et al., 2019a). For actinobacterial systematics, it is also critical to investigate the chemotaxonomic properties of new species across closely related taxa (Goodfellow et al., 2012). Developments in molecular systematics have been seen by some to question the continued significance of chemosystematics, but this view is unwarranted as the two approaches are complementary. The most valuable chemical characters are derived from analyses of cellular fatty acids, menaquinones, mycolic acids, muramic acid types, peptidoglycan types, polar lipids, and whole-organism sugars (Goodfellow, 1989). Some of these methods provide quantitative or semiquantitative data, as in the case of cellular fatty acid and menaquinone analyses, but others yield qualitative information, as exemplified by muramic acid, mycolic acid, peptidoglycan, phospholipid, and whole-organism sugar determinations.

Whole-genome sequencing provides the most comprehensive and reliable tool for comparative genome (Ekblom and Wolf, 2014). With the advent of whole-genome sequencing, the weakness of 16S rRNA gene sequencing is being compensated. The genome relatedness thresholds were established to determine whether the compared desert strains can be classified into the same taxon. The overall genome-related index (OGRI) is proposed to replace DNA–DNA hybridization for delineating new species (Chun et al., 2018). OGRI can be used to calculate the relatedness between the genome sequences of isolated strains and closely related strain types of a species. In this aspect, average nucleotide identity (ANI) and digital DDH (dDDH) are the two most widely used OGRI. The proposed and generally accepted species boundary for ANI and dDDH values are 95–96 and 70%, respectively (Goris et al., 2007; Richter and Rosselló-Móra, 2009; Meier-Kolthoff et al., 2013; Chun et al., 2018). These ANI and dDDH values represent a high correlation for the species delineation by a DDH cutoff value of 70% (Kim et al., 2014). Currently, genome sequences provided by Illumina (United States), Ion Torrent (Thermo Fisher Scientific, United States), and Pacific Biosciences (United States) platforms have been shown to generate high-quality DNA sequence data that meet the general standards for taxonomic purposes (Chun et al., 2018). It is evident that polyphasic taxonomy will be a standard approach for the taxonomic characterization of desert actinobacteria as it provides a reliable, reproducible, and stable delineation of taxa.

## Biotechnological Applications of Desert Actinobacteria

Desert actinobacteria generally undergo primary screening to have their interesting bioactivity profiles examined *in vitro* (Figure 4). For example, the obtained actinobacteria are subjected to a standard plug assay against a panel of wild-type microorganisms and *B. subtilis* reporter strains (Fiedler, 2004). Those exhibiting an extensive inhibition zone against Gram-positive and Gram-negative bacteria, fungi, and yeasts are assigned as potential antibacterial- or antifungal-producing strains. Similarly, plant beneficial traits, such as phytohormone production, siderophore production, and phosphate solubilization can be screened using qualitative assays to determine their agricultural potential (Allali et al., 2019). Potential actinobacteria are selected for the fermentation study of SMs followed by the purification of the obtained bioactive compounds. The chemical structure of the purified compounds will then be elucidated and tested for their biological properties. Desert actinobacteria with the unique ability to produce novel bioactive compounds could also be deposited as patent strains in public culture collections. In this section, we provide evidences for the potential applications of desert actinobacteria in agriculture, the environment, healthcare, and industry.

### Healthcare Applications

Actinobacteria isolated from the desert are potential sources of diverse SMs through their unique metabolic pathways, which adapt for survival under extreme habitats. Recently, the extreme biosphere is the focus of research on the discovery of bioactive compounds to combat MDR pathogens and diseases such as cancer, dementia, and epilepsy (Bull and Goodfellow, 2019). During the past two decades, more than 50 novel natural products have been isolated and identified from desert actinobacteria worldwide (Table 3) with potential antiallergic, antibacterial, anticytotoxic, antifungal, anti-inflammatory, antitumor, and antiviral activities. Particularly, members of the genus *Streptomyces* are responsible for more than half of the reported novel compounds.

**TABLE 3:** Novel natural products from desert actinobacteria between 2000 and 2021.

Organisms	Location	Structure class	Compound	Bioactivity	References
*Jiangella gansuensis* YIM 002^T^	Desert soil of Gansu, China	Pyrrol-2-aldehyde derivatives	Jiangrines A–E	Anti-inflammatory	Han et al., 2014, Jiao et al., 2017
		Glycolipid	Jiangolide		
		Indolizine derivative	Jiangrine F	Weak cytotoxicity	
*Lentzea chajnantorensis* H45^T^	Atacama Desert, Chile	New diene and monoene glycosides	Lentzeosides A–F	Anti-HIV-1 integrase activity	Wichner et al., 2017
*Nocardia* sp. XJ31	Xinjiang Desert, China	Ga-siderophores	Nocardimicins J–R	Virus polymerase inhibitory	Zhang et al., 2020
		Benz[α]anthraquinones	Brasiliquinone E	Anti-*Bacillus* Calmette–Guérin (BCG) activity	
*Saccharothrix algeriensis* SA 233^T^	Saharan soil, Algeria	Butanoyl-pyrrothine (BUP)	Dithiolopyrrolone antibiotics	Antibacterial and antifungal activities	Lamari et al., 2002, Zitouni et al., 2004, Strub et al., 2008
		Senecioyl-pyrothine (SEP)			
		Tigloyl-pyrrothine (TIP)			
*Saccharothrix algeriensis* NRRL B-24137		Crotonyl-pyrrothine	Dithiolopyrrolone PR2	Antibacterial, antifungal, and anti-yeast activities	Merrouche et al., 2011
		Sorbyl-pyrrothine	Dithiolopyrrolone PR8		
		2-Hexonyl-pyrrothine	Dithiolopyrrolone PR9		
		2-Methyl-3-pentenyl-pyrrothine	Dithiolopyrrolone PR10		
		Iso-hexanoyl-pyrrothine	Dithiolopyrrolone PR11		Merrouche et al., 2019
*Saccharothrix* sp. SA198		C_29_H_30_O_8_	Antibiotic A4	Antifungal and antibacterial activities	Boubetra et al., 2013a
		C_30_H_32_O_8_	Antibiotic A5		
*Streptomyces asenjonii* KNN 42. f	Atacama Desert, Chile	β-Diketones	Asenjonamides A–C	Antibacterial activity	Abdelkader et al., 2018
*Streptomyces leeuwenhoekii* C34^T^		New 22-membered macrolactone polyketides	Chaxalactins A–C	Antibacterial activity	Rateb et al., 2011b
		New ansamycin-type polyketides	Chaxamycins A–C	Anti-intrinsic ATPase activity of heat shock protein 90 (Hsp), anti-methicillin-resistant *Staphylococcus aureus* (MRSA), and antibacterials	Rateb et al., 2011a
			Chaxamycin D		
		Novel lasso peptide	Leepeptin	ND	Gomez-Escribano et al., 2019
*Streptomyces leeuwenhoekii* C38		New family of 22-membered antitumor macrolactones	Atacamycins A-C	Antibacterial, antitumor, and cytotoxic activities	Nachtigall et al., 2011
*Streptomyces leeuwenhoekii* C58		New lasso peptide	Chaxapeptin	Anti-human lung cancer cell line A549	Elsayed et al., 2015
*Streptomyces* sp. 8P21H-1	Taklamakan Desert, China	New streptogramin-type antibiotics	Acetyl-griseoviridin	No antibacterial activity	Wang et al., 2021
			Desulfurizing griseoviridin	Antibacterial activity	
*Streptomyces* sp. DA3-7	Saudi Arabian desert	New pyridine alkaloid	Pyridine alkaloid: pyridine-2,5-diacetamide	Antimicrobial activity	Nithya et al., 2018
*Streptomyces* sp. DB634	Atacama Desert, Chile	Aminoquinone derivatives	Abenquines A–D	Antibacterial, anti-dermatophytic fungal, and anti-phosphodiesterase type 4b activities	Schulz et al., 2011
*Streptomyces* sp. SAS02	Thar Desert, Rajasthan (India)	New anthracyclines	Non-named	Anticancer activity	Masand et al., 2018
*Streptomyces* sp. SAS09		New anthracycline glycoside		Antibacterial activity	
		New macrolide			
*Streptomyces* sp. SAS13		New anthracycline glycoside			
		New macrolide			
*Streptomyces* sp. SAS15		New macrolide			
					

#### Antibacterial and Antifungal Compounds

Diverse novel bioactive compounds produced from several desert actinobacteria have potential antibacterial and antifungal activities. A halotolerant actinobacterium, *J. gansuensis* YIM 002^T^, was isolated from a desert soil sample of Gansu. Its whole-genome analysis identified 60 functional gene clusters with the potential to produce pristinamycin, a known antibiotic effective for staphylococcal infections, and other antibiotics (Jiao et al., 2017). One new anthraquinone, brasiliquinone E produced by *Nocardia* sp. XJ31 from the Xinjiang Desert, demonstrated modest anti-*Bacillus* Calmette-Guérin (BCG) activity in an antituberculosis (anti-TB) assay (Zhang et al., 2020). The anti-TB activity indicated that brasiliquinone E can be used as a potential treatment for MDR TB. Three novel dithiolopyrrolone antibiotics, butanoyl-pyrrothine (BUP), senecioyl-pyrrothine (SEP), and tigloyl-pyrrothine (TIP), along with the known benzoyl-pyrrothine, iso-butyropyrrothine (ISP), and thiolutine, were produced from *S. algeriensis* SA 233^T^ (Lamari et al., 2002; Zitouni et al., 2004; Strub et al., 2008). Dithiolopyrrolones produced by this Saharan Desert (Algeria) strain were highly inhibitory against Gram-positive bacteria such as *Bacillus coagulans*, *B. subtilis*, and *Micrococcus luteus*. Three dithiolopyrrolones, ISP, and thiolutine are also effective against Gram-negative bacteria including *Klebsiella pneumoniae*. In addition, SEP and TIP showed greater activity against *S. cerevisiae*, *Mucor ramannianus*, and other phytopathogenic fungi (*Fusarium culmorum*, *Fusarium oxysporum* f.sp. *albedinis*, and *F. oxysporum* f.sp. *lini*), compared to thiolutine and ISP (Lamari et al., 2002). Two more Saharan Desert strains from Algeria are also capable of producing novel compounds. *Saccharothrix* strain SA198 generated two new antibiotics designated as A4 and A5, which inhibited both Gram-positive bacteria (*B. subtilis*, *Enterococcus faecalis*, and *Listeria monocytogenes*) and Gram-negative bacteria (*E. coli*, *K. pneumoniae*, and *Pseudomonas aeruginosa*) (Boubetra et al., 2013a). A4 and A5 also exhibited modest antifungal ability against filamentous fungi such as *Ascochyta fabae*, *Aspergillus carbonarius*, *F. culmorum*, *Fusarium equiseti*, *M. ramannianus*, and *Penicillium expansum*. By incorporating sorbic acid into its culture medium, *S. algeriensis* NRRL B-24137 produced five novel dithiolopyrrolone antibiotics: PR2, PR8, PR9, PR10, and PR11 (Merrouche et al., 2011, 2019). These five antibiotics showed antimicrobial activity against Gram-positive bacteria, filamentous fungi, and yeasts, including *B. subtilis*, *B. coagulans*, *L. monocytogenes*, *M. luteus*, *S. aureus*, *A. carbonarius*, *F. oxysporum* f.sp. *lini*, *Fusarium moniliforme*, *F. equiseti*, *F. culmorum*, *Fusarium graminearum*, *M. ramannianus*, *P. expansum*, *Candida albicans*, and *S. cerevisiae*.

Four strains obtained from the Atacama Desert were discovered to produce antibiotics with effective antibacterial and antifungal activities. *S. asenjonii* KNN 42.f generated asenjonamides A–C, three new bioactive β-diketones (Abdelkader et al., 2018). All compounds showed inhibitory activity against a panel of Gram-positive and Gram-negative bacteria, such as *S. aureus*, *B. subtilis*, *E. coli*, *E. faecalis*, and *Mycobacterium smegmatis*. Three new compounds named chaxalactins A–C (22-membered macrolactone polyketides), identified from *S. leeuwenhoekii* C34^T^, displayed strong inhibitory activity against Gram-positive bacteria (*S. aureus*, *L. monocytogenes*, and *B. subtilis*) and weak activity against Gram-negative bacteria (*E. coli* and *Vibrio parahaemolyticus*) (Rateb et al., 2011b). Another four new ansamycin-type polyketides, chaxamycins A–D, were shown to be active against *S. aureus*, *E. coli*, and a panel of methicillin-resistant *S. aureus* (MRSA) clinical isolates (epidemic MRSA and Scottish MRSA) (Rateb et al., 2011a). *S. leeuwenhoekii* C38 synthesized atacamycins A–C, three new 22-membered macrolactone antibiotics (Nachtigall et al., 2011). The antibacterial assay revealed that atacamycins were effective against Gram-positive and Gram-negative bacteria, including *B. subtilis*, *Brevibacterium epidermidis*, *Dermabacter hominis*, *K. pneumoniae*, *Propionibacterium acnes*, *P. aeruginosa*, *S. aureus*, *Staphylococcus epidermidis*, *Staphylococcus lentus*, and *Xanthomonas campestris*. Atacamycins A–C also slightly inhibited the growth of the phytopathogenic bacterium *Ralstonia solanacearum* DSM 9544 (Nachtigall et al., 2011). Four aminoquinone derivatives, abenquines A–D, isolated from *Streptomyces* sp. DB634, showed antibacterial and antifungal activities, in particular anti-dermatophytic fungi, against *Trichophyton rubrum*, *Trichophyton mentagrophytes*, and *Microsporum canis*, as well as slightly inhibiting *B. subtilis* and mouse fibroblasts (NIH-3T3 cell line) (Schulz et al., 2011).

Other desert actinobacterial strains also produced bioactive compounds, although their structures are unknown. *Streptomyces* sp. BS30 generated two undetermined compounds that are anti-*Aspergillus niger* 2CA936 and anti-yeast (Souagui et al., 2017). *Streptomyces* sp. Wb2n-11 has moderate antifungal, antibacterial, and antinematicidal activities against *F. culmorum*, *Rhizoctonia solani*, *Verticillium dahliae*, *R. solanacearum*, and root-knot nematode (*Meloidogyne incognita*) (Köberl et al., 2015). Several Thar Desert *Streptomyces* strains show bioactivity against MDR pathogens such as *C. albicans*, *E. coli* ATCC 3739, MRSA, *P. aeruginosa* ATCC 10145, and vancomycin-resistant *Enterococcus* (Masand et al., 2018). Three actinobacteria, *S. aburaviensis* Kut-8 (Thumar et al., 2010; Ramirez-Rodriguez et al., 2018), *S. asenjonii* KNN35.1b^T^ (Goodfellow et al., 2017), and *Yuhushiella* sp. TD-032 (Ibeyaima et al., 2016), were reported to inhibit Gram-positive bacteria including *S. aureus*, *Bacillus cereus*, *Bacillus megaterium*, and *B. subtilis*. *Micromonospora arida* LB32^T^ and *Micromonospora inaquosa* LB39^T^ were found to have antibacterial and antifungal abilities, especially against MDR *K. pneumoniae* ATCC 700603 (Carro et al., 2019a). *Nocardiopsis* sp. 38-7L-1 was able to treat the pathogen *P. aeruginosa* (Chen et al., 2015).

#### Anticancer and Antitumor Compounds

Chaxamycins A–D derived from *S. leeuwenhoekii* C34^T^ can be used as anticancer drugs based on their ability to inhibit the intrinsic ATPase activity of heat shock protein 90 (Hsp90) (Rateb et al., 2011a). Another strain, *S. leeuwenhoekii* C38, produced chaxapeptin, a new lasso peptide with a substantial inhibitory effect in the human lung cancer cell line A549 according to the cell invasion assay (Elsayed et al., 2015). Three antitumor compounds were also derived from *S. leeuwenhoekii* C38, atacamycins A–C, which moderately inhibited the enzyme phosphodiesterase (PDE-4B2) (Nachtigall et al., 2011). Furthermore, atacamycins A and B showed clear cytotoxic activities against a panel of 42 different human tumor cell lines. Atacamycin A was the most active in cell lines of colon cancer (CXF DiFi), breast cancer (MAXF 401NL), and uterus cancer (UXF 1138L), whereas atacamycin B showed significant antiproliferative activity against colon RKO cells (Nachtigall et al., 2011). *Micromonospora chalcea* LB4 and LB41 showed antitumor activity against human hepatocellular carcinoma (HepG2) cells (Carro et al., 2019a).

#### Anti-inflammatory Compounds

*Jiangella gansuensis* YIM 002^T^ was collected from Gansu Desert in China and produced seven new compounds, jiangrines A–F and jiangolide, as well as pyrrolezanthine, a known compound (Han et al., 2014). Jiangrines A–E and jiangrine F are pyrrol-2-aldehyde derivatives and an indolizine derivative, respectively. All of them, including pyrrolezanthine, demonstrated significant anti-inflammatory activity by inhibiting NO production in LPS-treated RAW 264.7 macrophage cells. Jiangolide is a glycolipid with weak cytotoxicity based on a low inhibitory ratio (<20%) of the cell viability assay (Han et al., 2014). Abenquines A–D isolated from *Streptomyces* sp. DB634 showed moderate anti-inflammatory activity against phosphodiesterase type 4 (PDE4b), which can be used to treat inflammatory diseases, such as chronic obstructive pulmonary disease (Schulz et al., 2011).

#### Antiviral Compounds

Lentzeosides A–F are six new diene and monoene glycosides derived from *Lentzea chajnantorensis* H45^T^ isolated from the Atacama Desert (Wichner et al., 2017). These novel compounds clearly inhibited HIV-1 integrase, one of the key enzymes in the HIV replication cycle. Thus, lentzeosides might be used as HIV integrase inhibitors for the treatment of HIV-1 infections, HIV-1 replication, and other virus strains resistant to multi-antiretroviral drugs (Wichner et al., 2017). *Nocardia* sp. XJ31, isolated from the Xinjiang Desert, generated nine new siderophores (nocardimicins J–R), which were found to inhibit viral infection by binding affinities with the four 3′′-RNA pockets of viral polymerases (>90%) (Zhang et al., 2020).

#### Antioxidant Compounds

*Streptomyces* sp. D25, isolated from Thar Desert, Rajasthan, showed antioxidant potential based on free radical scavenging activity using DPPH and nitric oxide assays (Radhakrishnan et al., 2016). It is also moderately effective against biofilm-forming bacteria, including *Alcaligenes* sp. M28, *Alcaligenes* sp. P8, *Bacillus* sp. M38, *Bacillus* sp. P13, *Kurthia* sp. P3, *Lactobacillus* sp. M6, *Lactobacillus* sp. M51, *Lactobacillus* sp. P4, *Micrococcus* sp. M50, *Pseudomonas* sp. P1, and *Staphylococcus* sp. M1.

### Agricultural Applications

Actinobacteria from the desert showed a broad range of plant growth-promoting and biocontrol properties. Their ability to survive under extreme environments allows them to enhance the growth of plants under severe abiotic stresses, especially drought and salinity. Since 2013, 37 actinobacteria from the desert have been reported to have agricultural potential, mostly from members of the genus *Streptomyces* as summarized in Table 4. All these studies highlight the potential application of desert actinobacteria in agriculture under stress environments.

**TABLE 4:** Agricultural applications from desert actinobacteria between 2000 and 2021.

Organism	Location	PGP traits	Target	References
*Arthrobacter* sp. AF3	Atacama Desert, Chile	Siderophore production, auxin (AIA) production, nitrogen fixation, ACC deaminase activity and phosphate solubilization	ND	Gaete et al., 2020
*Cryobacterium* sp. S5				
*Frondihabitans* sp. R8				
*Microbacterium* spp. M1-B and M2-A				
*Paeniglutamicibacter* spp. L1D and L2D				
*Pseudarthrobacter* spp. M1, M3, M2, and L2				
*Rhodococcus* sp. D4				
*Streptomyces* sp. M1-A				
*Cellulosimicrobium* sp. JZ28	Saudi Arabia, Jizan and AI Wahbah	Chitinase activity	Pathogens	Eida et al., 2020
		β-Glucosidases	Degradation of cellulose biopolymers	
		Osmoprotectants	Oxidative, osmotic, and salinity stresses	
		Volatiles: hydrogen sulfide	Biotic and abiotic stress	
*Kocuria turfanensis* 2M4	Saline desert of Little Rann of Kutch, Gujarat, India	IAA	Groundnut (*Arachis hypogaea* L.)	Goswami et al., 2014
*Microbacterium* sp. WLJ053	The rhizosphere of desert plant *Alhagi sparsifolia*	Plant growth-promoting activity	Maize	Wang et al., 2017
*Streptomyces* sp. WLJ079				
*Nocardiopsis dassonvillei* MB22	Sahara, Algeria	Biocontrol activity	Common root rot pathogen caused by *Bipolaris sorokiniana* LB12	Allali et al., 2019
		IAA, siderophore, hydrogen cyanide, chitinolytic activity, and solubilized inorganic phosphates	Durum wheat (cv. Vitron)	
*Streptomyces mutabilis* IA1	Saharan, Algeria	Biocontrol activity	Temperate crop	Toumatia et al., 2016
		IAA and GA3	Soft wheat (*Triticum aestivum* L.)	
*Streptomyces netropsis* A-ICA	Baja California, Mexico	Antifungal activity	*Macrophomina phaseolina*, *Fusarium oxysporum*, *Fusarium solani*, *Fusarium equiseti*, *Botrytis cinerea*, *Alternaria alternata*, and *Colletotrichum gloeosporioides*	Abdelmoteleb and González-Mendoza, 2020
		Inorganic phosphate solubilization	ND	
		IAA, GA	Tomato (*Solanum lycopersicum*)	
*Streptomyces* sp. AC5	Saudi Arabia, Jouf	Flavonoid, phytohormone, and siderophore production	Maize (*Zea mays* L.) under drought condition	Selim et al., 2019
*Streptomyces* sp. MM40	Monte Desert, NW of Patagonia, Neuquén	Auxin, cytokines, zeatins, and siderophores	ND	Solans et al., 2021
		Exoenzymes: proteases, phospholipase, and lipases		
*Streptomyces* sp. RD1 and RD5		Phosphorous solubilization	Native vegetation	
*Streptomyces* sp. MNB-1, MNB-2, MNB-3, MNC-1, MNC-2, MNC-3, MNC-4, MNT-1	Merzouga, Morocco	IAA, siderophore production, N_2_ fixation, P and K solubilization	ND	Nafis et al., 2019
*Streptomyces* sp. PT2	The Algerian Sahara	Crude IAA	Tomato	Goudjal et al., 2013
*Streptomyces* sp. UTMC 2482, UTMC 2483, and UTMC 3136	Salt Lake Qom, Iran	IAA, ethylene via ACC deaminase, and siderophores	Sunflower	Zahra et al., 2020
*Streptomyces* sp. Wb2n-11	Sinai Desert, Egypt	Antifungal	*Fusarium culmorum*; *Rhizoctonia solani*; *Verticillium dahliae*	Köberl et al., 2015
		Antibacterial	*Ralstonia solanacearum*	
		Antinematicidal	*Meloidogyne incognita* (soilborne phytopathogens)	

*ND, not determined.*

#### Plant Growth Promotion

Plant growth-promoting properties displayed by desert actinobacteria include 1-aminocyclopropane-1-carboxylic acid (ACC) deaminase production, auxin production, gibberellic acid (GA) production, indole-3-acetic acid (IAA) production, siderophore production, nitrogen fixation, and P and K solubilization. For example, a high-IAA-producing strain of *Kocuria turfanensis* 2M4 recovered from a saline desert in India significantly increased the total length and fresh biomass of groundnut after 15 days of germination by pot study (*Arachis hypogaea* L.) (Goswami et al., 2014). *Streptomyces mutabilis* IA1 from Saharan soil produced IAA and GA, which are responsible for the promotion of wheat growth after incubation in a phytotronic growth chamber for 10 days (Toumatia et al., 2016). *Nocardiopsis dassonvillei* MB22 was isolated from the Algerian Sahara soil and can stimulate the growth of durum wheat seedlings (shoot and root lengths and dry weight) by a variety of properties (chitinolytic activity, hydrogen cyanide production, IAA production, siderophore production, and inorganic phosphate solubilization) (Allali et al., 2019). Three plant growth-promoting *Streptomyces* isolated from the desert plant *Pteropyrum olivieri* could enhance the yield and biochemical contents of sunflower in greenhouse and field experiments under normal conditions (Zahra et al., 2020). These streptomycetes could also improve salt and drought tolerance in inoculated sunflower seedlings. In addition, they can act as plant probiotics by supplying the host plant with three major traits: acquisition of nutrients, growth hormone, and stress tolerance. *Microbacterium* sp. WLJ053 and *Streptomyces* sp. WLJ079 from the rhizosphere of *A. sparsifolia* harbored a nitrogen-fixation gene *nif*H, which improved maize growth by increasing stem and root lengths, fresh and dry weights after 1 month planting, and cultivation in the greenhouse (Wang et al., 2017). In addition, *S. netropsis* A-ICA from *Larrea tridentata* in the Baja California Desert of Mexico enhanced root elongation and development of wheat by the production of IAA, siderophores, and GA and phosphate solubilization after 4 days of germination in semisolid plates (Abdelmoteleb and González-Mendoza, 2020).

#### Mitigation Potential for Abiotic Stress in Plants

*Streptomyces* sp. AC5 from the semi-arid environment of Saudi Arabia could mitigate the negative effects of drought on the growth and physiology of maize, discovered from 6 weeks of cultivation in a controlled greenhouse (Selim et al., 2019). This IAA- and siderophore-producing *Streptomyces* sp. AC5 positively enhanced the growth and drought tolerance ability of maize by reducing H_2_O_2_ accumulation and lipid peroxidation through the production of antioxidants (total ascorbate, glutathione, tocopherols, phenolic acids, and flavonoids) and compatible solutes (sucrose, total soluble sugar, proline, arginine, and glycine betaine). The IAA and siderophores were suggested to promote root architecture and water and nutrient uptake from drought-affected soil (Selim et al., 2019). Similarly, *Cellulosimicrobium* sp. JZ28 was isolated from *Panicum turgidum* in the Saudi Arabia Desert, and its whole-genome sequence indicated that several genes were responsible for protecting the plant from environmental stresses, such as the ability to synthesize osmoprotectants and volatile compounds in order to withstand abiotic and biotic stresses (Eida et al., 2020).

#### Biocontrol Potential

*Streptomyces mutabilis* IA1 from Saharan soil exhibited biocontrol property to decrease severity (79.6%) and reduce occurrence (64.7%) of fungal infection caused by *F. culmorum* evaluated from 10 days young wheat (*Triticum aestivum* L.) seedlings in a phytotronic growth chamber (Toumatia et al., 2016). *N. dassonvillei* MB22, a powerful antifungal producing strain isolated from Algerian Sahara soil, could be used as a biocontrol agent to ensure crop health (Allali et al., 2019). This strain has the potential for controlling several soil-borne phytopathogens (*Bipolaris sorokiniana* LB12, *R. solani* LRS1, *F. culmorum* LF18, *F. graminearum* LF21 and *F. oxysporum* f.sp. *radices lycopersici* LF30). *Streptomyces* sp. UTMC 2482, UTMC 2483, and UTMC 3136 isolated from desert plant *P. olivieri* produced several lytic enzymes namely amylase, cellulase, chitinase, lipase and protease, which can inhibit growth of plant pathogens such as *A. niger*, *F. oxysporum* and *Mucor hiemalis* (Zahra et al., 2020). A rhizobacterium *S. netropsis* A-ICA from *Larrea tridentata* in the Baja California Desert of Mexico demonstrated high inhibitory activity on fungal growth (*Alternaria alternata*, *Botrytis cinerea*, *Colletotrichum gloeosporioides*, *F. equiseti*, *F. oxysporum*, *Fusarium solani*, and *Macrophomina phaseolina*) due to the production of hydrolytic enzymes (cellulases, chitinase, and glucanase) and antifungal compounds (Abdelmoteleb and González-Mendoza, 2020). The culture filtrate of *S. netropsis* A-ICA strongly suppressed the mycelial development of two fungal pathogens, *Botrytis cinerea* and *Macrophomina phaseolina*. The presence of chitinase genes suggested that *Cellulosimicrobium* sp. JZ28 isolated from *P. turgidum* in the Saudi Arabia Desert has a biocontrol potential against fungal pathogens, such as *B. cinerea*, *F. oxysporum* and *V. dahliae* (Eida et al., 2020).

### Industrial and Environmental Application

To adapt for living in the extreme arid ecosystems, various functional enzymes were synthesized by actinobacteria, and these enzymes can be applied for industrial and environmental uses. Those studies shown below provide supporting evidence for potential applications of desert actinobacteria in the environment and industry.

#### Pentachlorophenol Remediation

Pentachlorophenol (PCP) is a commonly utilized technique in some industries, such as the preservation of wood and leather (Kao et al., 2004). However, PCP is also toxic, and its exposure can induce acute pancreatitis, cancer, immunodeficiency, and neurological problems, as well as inhibiting oxidative phosphorylation (Sai et al., 2001; Shen et al., 2005). A halotolerant *Janibacter* sp. FAS23 strain obtained from arid and saline Tunisia terrain can degrade PCP (Khessairi et al., 2014). Strain FAS23 was able to degrade PCP up to 300 mg/L and could be used for PCP bioremediation in PCP-contaminated environments.

#### Industrial Potential

Two new oxidative enzymes, type I Baeyer-Villiger monooxygenases (BVMOs), industrial potential biocatalysts were discovered from *S. leeuwenhoekii* C34, isolated from Atacama Desert. These new BVMOs enzymes with high melting temperature (45°C) and water-miscible cosolvents tolerance were capable of NADPH-dependent BV oxidation activity on ketones, sulfoxidation activity on sulfides (Gran-Scheuch et al., 2018). In Saudi Arabia, *Streptomyces fragilis* DA7-7 synthesized a thermostable α-amylase, an important enzyme with commercial potential in industries, including brewery, detergent, paper, food, starch saccharification and pharmaceuticals. (Nithya et al., 2017). *Am*-fluorinase, another thermostable enzyme, was found in *A. mzabensis* from Saharan soil in Algeria (Sooklal et al., 2020). This enzyme is responsible for incorporating fluorine into organic molecules to improve the bioactive and biophysical characteristics of compounds and has been widely used in the agrochemical, material and pharmaceutical industries. The *Am*-fluorinase from *A. mzabensis* catalyzed a C-F bond and displayed great thermostability with the optimum temperature at 65°C. *Cellulosimicrobium* sp. JZ28, isolated from *P. turgidum* also contains genes encoding for β-glucosidases enzymes (Eida et al., 2020). This enzyme could be applied to extract the juice and liberate aroma from wine grapes by hydrolyzing bitter compounds, for example, it can enhance the flavor of fruit juice, tea, and wine in food processing industries (Singh et al., 2016).

## Conclusion and Future Perspectives

Diverse actinobacteria from the desert are undoubtedly potential resources for biotechnology. These actinobacteria which thrive under such extreme environmental conditions, exhibit the diversity and their exceptional adaptability from specialized metabolisms. This review explored 129 new species including alkaliphiles, halotolerant, thermophile and psychrotolerant from 35 deserts in the past two decades. Several species showed potential applications in biotechnology in particular bioactive compounds production, biocontrol properties, functional enzymes and plant growth promotion. The survival mechanisms of actinobacteria under extensive environmental stress in the desert offer tremendous possibilities to explore novel bioactive compounds with unique biosynthesis pathways. It is strongly supporting the view that gifted desert actinobacteria with their bioactive potential provide beneficial appropriate solutions to triumph over the increasingly serious threats from MDR pathogens, agricultural stress and environmental problems.

To recover untapped diversity of culturable actinobacteria from desert, it is extremely important and urgently need for develop improved techniques for selective isolation of target taxa. The progress of actinobacterial diversity depends on the development of such isolation methods which can reflect physicochemical characteristics of the desert environments. Growth of desert actinobacteria is mainly influenced by several physicochemical factors, such as the cation exchange capacity (CEC), the carbon and nitrogen sources, water retention capacity, hydrogen-ion concentration (pH) and temperature (Amin A. et al., 2020). To date, studies have revealed that the efficiency of actinobacterial isolation methods was positively related to the choices of selective media, supplemented inhibitors, inoculation techniques and incubation conditions, which mimics desert environments (Goodfellow, 2010; Goodfellow and Fiedler, 2010; Tiwari and Gupta, 2013). Selective media provide nutrient sources require for the growth and proliferation of target actinobacteria with the help of inhibitors to suppress the growth of unwanted bacteria. Most desert actinobacteria are slow-growing actinobacteria, with a preference for poor nutrient media over nutrient rich media (Li et al., 2021). The supplement of selective antibiotics was significantly promoted the isolation of desert taxa by sharply reduced the number of unwanted bacteria represented by the genus *Bacillus*. Media supplemented with these antibiotics far exceeded in obtaining the target actinobacteria than media without supplementation (Li et al., 2021). In addition, a higher number of isolates were recorded from the isolation media using the sprinkling technique for inoculation as compared with those corresponding plates prepared from the traditional serial dilution method (Idris, 2016; Xie, 2017). As opposed to the isolation plates seeded with soil suspensions, sprinkling technique allowed the mineral particles to be in direct contact with the nutrients in the media, which may stimulate spore germination and improve the growth of actinobacteria. More diversity was recovered from mineral particles inoculated onto selective media supplemented with inhibitor using sprinkling technique as exemplified by Idris (2016) and Xie (2017). Therefore, it is strongly recommended to use the sprinkling technique for the isolation of actinobacteria from desert associated samples.

In addition to culture-dependent studies, next-generation sequencing techniques and metagenomics are widely used in the identification and characterization of actinobacteria to gain an understanding of their diversity and functions in the desert. However, most desert microorganisms cannot be cultured as exemplified by only 1% of them are culturable (Martiny, 2019). Whole-genome sequences seem to be an effective way to resolve these taxonomic bottlenecks with increasing utilization in systematic studies. It provides a deep insight into the ecology and biotechnological potential of desert species. Currently, 16,763 reference genomes are available in the database (Shi et al., 2021). With the increasing availability of genome sequences, the information regarding genes and their functions provides insights into the metabolic potential of desert actinobacteria, which will greatly promote their biotechnology applications. A culture-enriched metagenomic approach named PLate Coverage Algorithm (PLCA) is developed based on the use of 16S rRNA gene sequencing of culture-enriched plates and shotgun metagenomics to identify the microbial community and their functions, which has been succeeded to recover bacteria including actinobacteria from the cystic fibrosis (Whelan et al., 2020). The PLCA improves the recovery of microbial diversity in the sample and provides a better understanding of the mechanisms between microbial communities and the host environment. This method can be used on any low-abundance microbial community in particular low-abundance actinobacterial taxa from desert, such as *Desertiactinospora*, *Tenggerimyces*. Another method for recovering unculturable actinobacteria is the cooperation of 16S rRNA gene metagenomics and commercially available media (Ito et al., 2019). These authors suggested that selective features of media are significantly influenced the recovery of slow-growing taxa on the isolation plates. Successful isolation of novel actinobacteria from commercially available media suggested that uncultured actinobacteria are potentially culturable. This method highlights the high possibility of the recovery of low-abundance taxa in laboratory culture. Besides, computational techniques are continuedly developed since several new algorithms, tools and databases are becoming available, aiming to explore both actinobacterial diversity, their functions and biotechnological potential (Medema, 2021). Several bioinformatics tools are designed specifically for the prediction of bioactive compounds with novel chemical structures from the whole genome sequences (Mikulak-Klucznik et al., 2020; Stokes et al., 2020). An effective biosynthetic pathway for such new compounds can be selected and mined through these genomic data for further engineering purposes. These exciting improvements will undoubtedly provide a tool for the recovery of novel taxa, their ecological functions and the discovery of un-hitherto biotechnological values of these desert actinobacteria. The more comprehensive knowledge should be built upon the principle of “embrace the genome” (Whitman, 2014), which would provide an immensely richer understanding of the biology of actinobacteria in desert environments.

## Author Contributions

FX wrote the first draft. WP conceived the idea and supervised FX. FX and WP revised the manuscript. Both authors contributed to the article and approved the submitted version.

## Conflict of Interest

The authors declare that the research was conducted in the absence of any commercial or financial relationships that could be construed as a potential conflict of interest.

## Publisher’s Note

All claims expressed in this article are solely those of the authors and do not necessarily represent those of their affiliated organizations, or those of the publisher, the editors and the reviewers. Any product that may be evaluated in this article, or claim that may be made by its manufacturer, is not guaranteed or endorsed by the publisher.
