# Supplementary material for: Actinobacteria From Desert: Diversity and Biotechnological Applications
Source: Front Microbiol. 2021 Dec 9;12:765531. doi: 10.3389/fmicb.2021.765531 (PMC8696123; doi:10.3389/fmicb.2021.765531)
Supplement: Supplementary file 1 [file Data_Sheet_1.docx]

Supplementary Material

**Supplementary Table 1.** Regions and locations of novel desert actinobacteria from 2000 to 2021.

| **Region** | **Location** | **Organisms** |
| --- | --- | --- |
| Central Africa | Sahara Desert, Chad | *Geodermatophilus africanus* CF11/1^T^; *Geodermatophilus arenarius* CF5/4^T^; *Geodermatophilus saharensis* CF5/5^T^; *Geodermatophilus siccatus* CF6/1^T^; *Geodermatophilus telluris* CF9/9/9^T^; *Geodermatophilus tzadiensis* strains CF5/2^T^, CF5/2 and CF7/1 |
| North Africa | Desert soil from Beni-Suef Governorate, Egypt | *Nocardiopsis benisuefensis* WS65 |
|  | Eastern Desert of Egypt | *Citricoccus alkalitolerans* YIM 70010^T^; *Dietzia lutea* YIM 80766^T^; *Nocardiopsis alkaliphila* YIM 80379^T^; *Streptomyces sannurensis* WS 51^T^ |
|  | Saline and alkaline desert in Egypt | *Kocuria aegyptia* YIM 70003^T^ |
|  | Béni-Abbès, Béchar, Algeria | *Streptosporangium becharense* SG1^T^ |
|  | Béni-Isguen (Mzab), Algeria | *Prauserella isguenensis* strains H225^T^ and H137 |
|  | Sahara Desert, Algeria | *Actinophytocola algeriensis* MB20^T^; *Actinopolyspora mzabensis* H55^T^; *Saccharothrix algeriensis* SA 233^T^; *Saccharothrix ghardaiensis* MB46^T^; *Saccharothrix hoggarensis* SA181^T^; *Saccharothrix isguenensis* MB27^T^; *Saccharothrix tamanrasssetensis* SA198^T^; *Streptosporangium algeriense* 169^T^ |
|  | Sahara Desert, Libya | *Geodermatophilus pulveris* BMG 825^T^ |
|  | Arid and saline sediment, Tunisia | *Janibacter* sp. FAS23 |
|  | Sahara Desert, Tunisia | *Geodermatophilus sabuli* BMG 8133^T^; *Promicromonospora panici* PT9^T^ |
| South Africa | Namib Desert, Namibia | *Actinomadura namibiensis* HAG 010767^T^ |
| North America | Baja California Desert, Mexico | *Streptomyces netropsis* strain A-ICA |
| South America | Atacama Desert, Chile | *Amycolatopsis ruanii* NMG112^T^; *Amycolatopsis thermalba* SF45^T^; *Amycolatopsis vastitatis* H5^T^; *Blastococcus atacamensis* P6^T^; *Geodermatophilus chilensis* B12^T^; *Lechevelieria atacamensis* C61^T^; *Levhevalieria deserti* C68^T^; *Lechevalieria roselyniae* C81^T^; *Lentzea chajnantorensis* H45^T^; *Micromonospora acroterricola* 5R2A7^T^; *Micromonospora arida* LB32^T^; *Micromonospora inaquosa* LB39^T^; *Modestobacter altitudinis* 1G4^T^; *Modestobacter caceresii* KNN 45-2b^T^; *Modestobacter excelsi* 1G6^T^; *Pseudonocardia nigra* strains ATK03^T^, ATK01 and ATK17 (rock); *Streptomyces altiplanensis* HST21^T^; *Streptomyces aridus* H9^T^; *Streptomyces asenjonii* KNN35.1b^T^; *Streptomyces atacamensis* C60^T^; *Streptomyces deserti* C63^T^; *Streptomyces bullii* C2^T^; *Streptomyces leeuwenhoekii* strains C34^T^, C38, C58 and C79. |
| Central Asia | Karakum Desert, Turkmenistan | *Desertiactinospora gelatinilytica* 7K107^T^; *Jiangella asiatica* 5K138^T^; *Jiangella aurantiaca* 8K307^T^; *Jiangella ureilytica* KC603^T^; *Kribbella turkmenica* 16K104^T^; *Micromonospora deserti* 13K206^T^; *Nonomuraea deserti* KC310^T^; *Nonomuraea diastatica* KC712^T^; *Nonomuraea longispora* KC201^T^; *Nonomuraea mesophila* 6K102^T^; *Streptomyces cahuitamycinicus* 13K301^T^ |
| East Asia | Badain Jaran Desert, China | *Cellulomonas telluris* CPCC 204705^T^; *Desertihabitans aurantiacus* CPCC 204711^T^ |
|  | Colour Desert of Tibet Autonomous, China | *Streptomyces dengpaensis* XZHG99^T^ |
|  | Desert soil of Gansu, China | *Jiangella gansuensis* YIM 002^T^ |
|  | Desert soil of Hangjin Banner, Ordos, Inner Mongolia, Northern China | *Kribbella deserti* SL15-1^T^ (rhizosphere soil) |
|  | Desert soil in Isolaginsha, China | *Lentzea isolaginshaensis* NX62^T^ |
|  | Desert soil of Xinjiang, China | *Actinomadura deserti* BMP B8004^T^; *Kineococcus xinjiangensis* S2-20^T^; *Nocardia* sp. XJ31; *Ornithinicoccus halotolerans* EGI 80423^T^; *Prauserella shujinwangii* XJ46^T^; *Saccharothrix deserti* B8144^T^; *Yuhushiella deserti* RA45^T^ |
|  | Gurbantunggut Desert, China | *Blastococcus deserti* SYSU D8006^T^; *Desertimonas flava* SYSU D60003^T^; *Microbacterium halophytorum* strains YJYP 303^T^ and YZYP 518; *Microbacterium suaedae* YZYP 306^T^; *Streptomyces desertarenae* SYSU D8023^T^ |
|  | Saline-alkaline desert soil of Fukang, China | *Nesterenkonia rhizosphaerae* EGI 80099^T^ (rhizosphere soil); *Streptomyces fukangensis* EGI 80050^T^ |
|  | Taklamakan Desert, China | **Aeromicrobium endophyticum* 9W16Y-2^T^; *Desertihabitans brevis* 16Sb5-5^T^; **Labedella phragmitis* 11W25H-1^T^; **Labedella populi* 8H24J-4-2^T^; **Microbacterium karelineae* TRM 80801^T^; *Mycetocola manganoxydans* MB1-14^T^; *Nakamurella deserti* 12Sc4-1^T^ (rhizosphere soil); *Nesterenkonia populi* GP10-3^T^; *Nocardioides deserti* SC8A-24^T^ (rhizosphere soil); *Nocardioides vastitatis* 21Sc5-5^T^; **Nocardiopsis* sp. 38-7L-1 (*Psammophytes*); *Planctomonas deserti* 13S1-3^T^; *Prauserlla endophytica* SP28S-3^T^; *Streptomyces taklimakanensis* TRM43335^T^ |
|  | Tengger Desert, China | *Actinophytocola gilvus* I12A-02593^T^; *Tenggerimyces mesophilus* I12A-02601^T^ |
|  | Turpan Desert, China | *Actinoplanes deserti* YIM CF22^T^; *Aeromicrobium halotolerans* YIM Y47^T^; *Arthrobacter deserti* YIM CS25^T^ |
| South Asia | Kunjam Pass, a cold desert of Indian Himalayas | *Dietzia kunjamensis* K30-10^T^ |
|  | Lahual-Spiti Valley, a cold desert of Indian Himalayas | *Actinoalloteichus spitiensis* RMV-1378^T^; *Agrococcus lahaulensis* K22-21^T^; *Kocuria himachalensis* K07-05^T^; *Ornithinimicrobium kibberense* K22-20^T^; *Rhodococcus kroppenstedtii* K07-23^T^ |
|  | Saline desert of Kutch, India | *Streptomyces aburaviensis* strain Kut-8 |
|  | Thar Desert, India | *Saccharothrix tharensis* TD-093^T^; *Yuhushiella* sp. TD-032 |
|  | Cholistan Desert, Pakistan | *Auraticoccus cholistanensis* F435^T^; *Arthrobacter mobilis* E918^T^; *Motilibacter aurantiacus* K478^T^; *Motilibacter deserti*  E257^T^ |
|  | Thal Desert in Punjab, Pakistan | *Nocardioides thalensis* NCCP-696^T^ |
| Middle East | Anatolia, Turkey | *Nonomuraea terrae* CH32^T^ |
|  | Arid area, Saudi Arabia | *Georgenia alba* SYSU D8008^T^; *Georgenia deserti* SYSU D8004^T^; *Microbacterium album* SYSU D8007^T^; *Microbacterium deserti* D8014^T^; *Saccharopolyspora deserti* D8010^T^; *Streptomyces fragilis* DA7-7 |
| South Pole | Desert soils, Antarctic | *Modestobacter multiseptatus* AA-826^T^ (rock, Transantarctic Mountains); *Williamsia* sp. D3 (Darwin Mountains) |
| Oceania | Arid Australian soils | *Amycolatopsis australiensis* GY048^T^; *Amycolatopsis deserti* GY024^T^; *Amycolatopsis granulosa* GY307^T^; *Amycolatopsis thermophila* GY088^T^; *Amycolatopsis viridis* GY115^T^ |

Key: *: Species isolated from plants in desert area; T: type strain.

**Supplementary Table 2.** Primers used for molecular identification of desert actinobacteria.

| **Serial number** | **Name of primers** | **Reference** |
| --- | --- | --- |
| 1 | PA (5'-CAGAGTTTGATCCTGGGCT-3') forward primer | Li, L. et al., 2019b |
|  | PB (5'-AGGAGGTGATCCAGCCGCA-3') reverse primer |  |
| 2 | PB36 (5'-AGRGTTTGATCMTGGCTCAG -3') forward primer | Guerrero et al., 2014 |
|  | PB38 (5'-GKTACCTTGTTACGACTT -3') reverse primer |  |
| 3 | Tot-F2 (5'-TCCTACGGGAGGCAGCAGT-3') forward primer | Abdelmoteleb et al., 2020 |
|  | Tot-R2 (5'-GGACTACCAGGGTATCTAATCCTGTT-3') reverse primer |  |
| 4 | COM2xF (5'-AAACTCAAAGGAATTGACGG-3') forward primer | Idris et al., 2017b |
|  | AC1186R (5'-CTTCCTCCGAGTTGACCC-3') reverse primer |  |
| 5 | 8-27F (5'-CCGTCGACGAGCTCAGAGTTTGATCCTGGCTCAG-3') forward primer | Cui et al., 2001 |
|  | 1523-1504R (5'-CCCGGGTACCAAGCTTAAGGAGGTGATCCAGCCGCA-3') reverse primer |  |
| 6 | 8-27F (5'-CCGTCGACGAGCTCAGAGTTTGATCCTGGCTCAG-3') forward primer | Meklat et al., 2013 |
|  | 1541-1522R (5'-AAGGAGGTGATCCAGCCGCA-3') reverse primer |  |
| 7 | 8-27F (5'-CCGTCGACGAGCTCAGAGTTTGATCCTGGCTCAG-3') forward primer | Mayilarj et al., 2006d |
|  | 1500R (5'-AGAAAGGAGGTGATCCAGGC-3') reverse primer |  |
| 8 | 27F (5'-AGAGTTTGATC(AC)TGGCTCAG-3') forward primer | Saygin et al., 2019a; Saygin et al., 2020a, b, c |
|  | 1492R (5'-ACGG(CT)TACCTTGTTACGACTT-3') reverse primer |  |
| 9 | 27F (5'-AGAGTTTGATCMTGGCTCAG-3') forward primer | Carro et al., 2019a |
|  | 1522R (5'-AAGGAGGTGATCCANCCRCA-3') reverse primer |  |
| 10 | 27F (5'-AGAGTTTGATCMTGGCTCAG-3') forward primer | Tan, G. Y. et al., 2006 |
|  | 1525R (5'-AAGGAGGTGWTCCARCC-3') reverse primer |  |
| 11 | 63F (5'-CAGGCCTAACACATGCAAGTC-3') forward primer | Belov et al., 2019 |
|  | 1387R (5'-GGGCGGWGTGTACAAGGC-3') reverse primer |  |
