# Supplementary material for: Actinobacteria From Desert: Diversity and Biotechnological Applications
Source: Front Microbiol. 2021 Dec 9;12:765531. doi: 10.3389/fmicb.2021.765531 (PMC8696123; doi:10.3389/fmicb.2021.765531)
Supplement: Supplementary file 2 [file Table_3.docx]

**Supplementary Table 3.** Selective isolation with incubation conditions of desert actinobacteria between 2000 and 2021.

| **Genus** | **Selective media** | **Antibiotics** | **Incubation period** | **Optimum growth temperature** | **Reference** |
| --- | --- | --- | --- | --- | --- |
| *Actinoalloteichus* | Actinomycetes isolation agar | - | - | 25 °C | Singla et al., 2005 |
| *Actinomadura* | Humic acid-vitamin (HV) agar | Cycloheximide (20 mg/l), nalidixic acid (10 mg/l) and nystatin (20 mg/l) | 21 days | 28 °C | Cao et al., 2018 |
|  | Actinomycetes isolation agar | - | - | - | Wink et al., 2003 |
| *Actinophytocola* | Chitin-vitamin agar | Cycloheximide (80 µg/ml) | - | 20 - 40 °C | Bouznada et al., 2016b |
|  | Glycerol-asparagine agar (ISP 5) | - | 3 weeks | 28 °C | Sun et al., 2014 |
| *Actinoplanes* | Tryptose soya agar | Nystatin (100 mg/l) | 2 weeks | 37 °C | Habib et al., 2018 |
| *Actinopolyspora* | HV agar + 20 % (w/v) NaCl | Actidione (50 µg/ml) | 25 days | 30 °C | Meklat et al., 2013 |
| *Aeromicrobium* | Modified Gauze’s medium No. 2 | - | 2 - 4 weeks | 28 - 37 °C | Li, F. et al., 2019a |
|  | Tryptone soya agar | Nystatin (100 mg/l) | 2 weeks | 30 °C | Yan et al., 2016 |
| *Agrococcus* | Tryptic soy agar (TSA) | - | - | 30 °C | Mayilraj et al., 2006e |
| *Amycolatopsis* | SM2 agar | Neomycin (4 µg/ml), D (+) melezitose (1%, w/v) and nystatin (50 µg/ml) | 3 weeks | 28 - 45 °C | Tan et al., 2006 |
|  | SM1 agar | Neomycin (1 µg/ml), cycloheximide and nystatin (each at 25 µg/ml) | 3 weeks | 45 °C | Busarakam et al., 2016a |
|  | SM2 agar | Neomycin (4 µg/ml), D (+) melezitose (1%, w/v) and nystatin (50 µg/ml) | 21 days | 20 - 50 °C | Zucchi et al., 2012a |
|  | SM3 agar | Cycloheximide (50 µg/ml), nalidixic acid (10 µg/ml), novobiocin (10 µg/ml) and nystatin (50 µg/ml) |  |  |  |
|  | Starch-casein agar | - |  |  |  |
|  | SM1 agar | Neomycin (4 µg/ml), D (+) sorbitol (1%, w/v) and nystatin (50 µg/ml) |  | 10 - 50 °C | Zucchi et al., 2012b |
|  | SM2 agar | Neomycin (4 µg/ml), D (+) melezitose (1%, w/v) and nystatin (50 µg/ml) |  |  |  |
|  | Glucose-yeast extract agar | Cycloheximide and nystatin (each at 25 µg/ml) | 14 days | 35 °C | Idris et al., 2018 |
| *Auraticoccus* | Glucose-yeast extract-malt agar (ISP 2) | Cycloheximide (45 mg/l), potassium dichromate (45 mg/l) and nalidixic acid (25 mg/l) | 7-14 days | 25 - 30 °C | Cheema et al., 2020 |
| *Arthrobacter* | TSA medium | Nystatin (100 mg/l) | 2 weeks | 28 °C | Hu et al., 2016 |
|  | ISP 2 agar | Nalidixic acid (20 mg/l) | 2 – 4 weeks |  | Ye et al., 2020 |
| *Blastococcus* | GYM *Streptomyces* (DSMZ medium No. 65) | Nalidixic acid (10 µg/ml), cycloheximide and nystatin (each at 25 µg/ml) | 2 weeks | 35 °C | Castro et al., 2018a |
|  | Reasoner’s 2A (R2A) agar | - | 7 days | 28 °C | Yang et al., 2019 |
| *Cellulomonas* | Modified cellulose-casein-multisalts (CCMS) agar | - | 3 weeks | 28 °C | Shi et al., 2020 |
| *Citricoccus* | Medium A | - | 1 weeks | 28 °C | Li, W. et al., 2005 |
| *Desertiactinosopra* | *Nocardia* agar | Cycloheximide (50 mg/ml) and rifampicin (5 µg/ml) | 28 days | 28 °C | Saygin et al., 2019a |
| *Desertihabitans* | Yeast-potassium hydrogen phosphate medium | Nystatin (50 mg/l) and potassium dichromate (30 mg/l) | 21 days | 28 °C | Sun, Y. et al., 2019 |
|  | HV agar | Cycloheximide (45 mg/l), nalidixic acid (25 mg/l) and potassium dichromate (45 mg/l) | 4 weeks | 37 °C | Liu, S. et al., 2020a |
| *Desertimonas* | 0.1 × Trypticase soy agar | - | 30 days | 28 °C | Asem et al., 2018 |
| *Dietzia* | Tryptic soy agar (TSA) | - | - | 25 °C | Mayilarj et al., 2006d |
|  | Horikoshi I medium |  | 2 weeks | 28 °C | Li et al., 2009 |
| *Geodermatophilus* | R2A (DSMZ medium 830) | - | 3 – 10 days | 25 - 35 °C | Montero-Calasanz et al., 2013b |
|  | Trypticase soy broth (TSB; DSMZ medium 535) |  |  |  |  |
|  | R2A and TSA agar |  |  | 28 - 40 °C | Montero-Calasanz et al., 2012 |
|  |  |  |  | 20 - 35 °C | Montero-Calasanz et al., 2013c ; 2013d |
|  |  |  |  | 20 - 37 °C | Montero-Calasanz et al., 2013e |
|  |  |  |  | 15 - 40 °C | Montero-Calasanz et al., 2013a |
|  | *Geodermatophilus obscurus* agar | Nalidixic acid (10 µg/ml), cycloheximide and nystatin (each at 25 µg/ml) | 2 weeks | 28 °C | Castro et al., 2018b |
|  | Luedemann medium (DSMZ medium 877) | - | 3 weeks | 30 - 35 °C | Hezbri et al., 2016 |
|  | R2A agar |  |  |  |  |
|  | Luedemann medium (DSMZ medium 877) |  | 15 days | 35 - 40 °C | Hezbri et al., 2015 |
| *Georgenia* | R2A agar | - | 5 days | 28 - 37 °C | Hozzein et al., 2018 ;  Li, L. et al., 2019b |
| *Jiangella* | HV agar | - | 28 days | 20 - 37 °C | Saygin et al., 2020b |
|  | *Nocardia* agar |  |  | 28 - 37 °C |  |
|  | Reasoner’s 2A (R2A) agar |  |  |  |  |
|  | Glycerol-asparagine agar (ISP 5) |  | 2 weeks | 28 °C | Jiao et al., 2017 |
| *Janibacter* | Yeast extract-mannitol medium (YEM) | - | 7 days | 30 °C | Khessairi et al., 2014 |
| *Kineococcus* | Tenfold-diluted TSB agar | - | 7 days | 37 °C | Liu, M. et al., 2009 |
| *Kocuria* | Horikoshi agar medium | - | - | 28 °C | Li, W. et al., 2006 |
|  | TSA medium |  |  | 30 °C | Mayilraj et al., 2006b |
| *Kribbella* | Luria-Bertani (LB) agar | - | 2 - 3 days | 30 °C | Sun et al., 2017 |
|  | Chitin-vitamin agar | Cycloheximide (50 µg/ml) and nalidixic acid (10 µg/ml) | 15 days | 28 °C | Saygin et al., 2019b |
| *Labedella* | Chitin medium | Cycloheximide (50 mg/l), nalidixic acid (20 mg/l) and potassium dichromate (50 mg/l) | 4 – 8 weeks | 28 - 30 °C | Li, F. et al., 2019b |
|  | Gauze’s medium No. 2 |  |  | 30 - 32 °C |  |
| *Lechevalieria* | SM2 agar | Neomycin (4 µg/ml), D (+) melezitose (1%, w/v) and nystatin (50 µg/ml) | 21 days | 30 °C | Okoro et al., 2010 |
| *Lentzea* | Tyrosine agar (ISP 7) | K_2_Cr_2_O_7_ (25 mg/l), calcium propionate (30 mg/ml) and cycloheximide (50 mg/ml) | 5 days | 20 - 40 °C | Wang et al., 2019 |
|  | Gauze’s No. 1 agar | Cycloheximide and nystatin (each at 25 µg/ml) | 14 days | 28 °C | Idris et al., 2017c |
| *Microbacterium* | R2A agar | - | 5 days | 28 °C | Yang et al., 2018b |
|  | Oatmeal agar (ISP 3) and Glycerol-asparagine agar (ISP 5) |  | 2 weeks |  | Li, Y. R. et al., 2018;  Zhu et al., 2019 |
| *Micromonospora* | R2A agar | Cycloheximide and nalidixic acid (each at 50 µg/ml) | 4 weeks | 28 °C | Carro et al., 2019b |
|  | Starch-casein agar | Cycloheximide, nystatin and novobiocin (each at 25 µg/ml) | 3 weeks |  | Carro et al., 2019a |
|  | Minimal medium | Cycloheximide (50 µg/ml) and nalidixic acid (20 µg/ml) | 28 days |  | Saygin et al., 2020a |
| *Modestobacter* | Gauze’s No. 1 agar | Cycloheximide and nystatin (each at 50 µg/ml) | 21 days | 28 °C | Golińska et al., 2020a; 2020b |
|  | HV agar | Actidione (25 µg/ml) and nalidixic acid (10 µg/ml) | 3 weeks | 28 °C | Busarakam et al., 2016b |
|  | Oligotrophic medium PYGV | - | 1 month | 19 - 21 °C | Mevs et al., 2000 |
| *Motilibacter* | Yeast-malt extract agar | Cycloheximide (45 mg/l), nalidixic acid (25 mg/l) and potassium dichromate (45 mg/l) | 4 – 8 weeks | 37 °C | Liu, S. et al., 2020c |
| *Mycetocola* | Modified 0.3 × marine broth agar | - | 1 week | 30 °C | Luo et al., 2012 |
| *Nakamurella* | TSA medium | Cycloheximide (45 mg/l), nalidixic acid (25 mg/l) and potassium dichromate (45 mg/l) | 8 weeks | 28 - 30 °C | Liu, S. et al., 2019a |
| *Nesterenkonia* | Glucose peptone (GP) agar | - | 6 weeks | 37 °C | Liu, J. et al., 2015b |
|  | Glycerol-asparagine agar |  | - | 10 - 45 °C | Wang et al., 2014 |
| *Nocardia* | HV agar, SM3 agar, MOPS agar, Starch-casein agar and Arginine vitamins (AV) agar | - | 7 – 14 days | 28 °C | Zhang et al., 2020 |
| *Nocardioides* | Modified starch casein agar | - | 8 weeks | 30 °C | Tuo et al., 2015 |
|  | R2A agar |  | 7 days | 30 - 37 °C | Khan et al., 2017 |
|  |  | Cycloheximide (45 mg/l), nalidixic acid (25 mg/l) and potassium dichromate (45 mg/l) | 8 weeks | 28 - 30 °C | Liu, S. et al., 2020 |
| *Nocardiopsis* | Medium A | - | 14 days | 28 - 30 °C | Hozzein et al., 2004 |
| *Nonomuraea* | Stevenson’s medium No. 3 (SM3) | Cycloheximide (50 µg/ml) and nalidixic acid (10 µg/ml) | 28 days | 28 °C | Saygin et al., 2020c |
|  | Marine agar |  |  |  |  |
|  | R2A agar |  |  |  |  |
|  | M1 agar |  |  |  |  |
|  | Czapek-Dox agar | Nystatin (50 µg/ml) and rifampicin (5 µg/ml) | 3 weeks | 30 °C | Ay, 2020 |
| *Ornithinicoccus* | R2A agar | - | 3 weeks | 30 °C | Zhang et al., 2016 |
| *Ornithinimicrobium* | Tryptone soya agar medium | - | - | 28 °C | Mayilraj et al., 2006c |
| *Planctomonas* | HV agar | Cycloheximide (45 mg/l), nalidixic acid (25 mg/l) and potassium dichromate (45 mg/l) | 4 weeks | 28 - 30 °C | Liu, S. et al., 2019b |
| *Prauserella* | SP agar | - | 4 weeks | 28 - 37 °C | Liu, J. et al., 2015a |
|  | Chitin- vitamin agar + 15% (w/v) NaCl | Cycloheximide (50 mg/l) | - | 30 - 37 °C | Saker et al., 2015 |
|  | SCN agar | - | 2 weeks | 16 - 45 °C | Liu et al., 2014 |
| *Promicromonospora* | TSA medium | - | 7 days | 30 °C | Guesmi et al., 2021 |
| *Pseudonocardia* | Yeast extract-malt extract agar | - | 4 weeks | 10 - 30 °C | Trujillo et al., 2017 |
| *Rhodococcus* | TSA medium | - | - | 30 °C | Mayilarj et al., 2006a |
| *Saccharopolyspora* | R2A agar + 5% (w/v) NaCl | - | 7 days | 28 - 37 °C | Yang et al., 2018a |
| *Saccharothrix* | Humic acid/B vitamin agar | Streptomycin sulphate (10 µg/ml) and actidione (50 µg/ml) | - | 18 - 45 °C | Zitouni et al., 2004 |
|  | Oatmeal agar | Nalidixic acid (10 mg/l), nystatin (20 mg/l) and cycloheximide (20 mg/l) | 28 days | 28 °C | Liu, J. et al., 2020 |
|  | Chitin-vitamin agar | Actidione (50 µg/ml) | - | 30 °C | Boubetra et al., 2015 |
|  |  | Cycloheximide (80 µg/ml) |  |  | Bouznada et al., 2017 |
|  | HV agar | Actidione (50 µg/ml) |  |  | Boubetra et al., 2013b |
|  |  | Cycloheximide (80 µg/ml) |  |  | Bouznada et al., 2016a |
|  | Starch-casein agar | Cycloheximide (25 µg/ml) | 10 days |  | Ibeyaima et al., 2018 |
| *Streptomyces* | Starch-casein agar (SCA) | - | 6 days | 30 °C | Thumar et al., 2010 |
|  | Glucose-yeast extract agar | Cycloheximide and nystatin (each at 25 µg/ml) | 14 days | 28 °C | Idris et al., 2017b |
|  | HV agar | - |  | 37 °C | Goodfellow et al., 2017 |
|  | SCA medium | Cycloheximide and nystatin (each at 50 µg/ml) | 14-21 days | 28 -37 °C | Cortés-Albayay et al., 2019 |
|  | Raffinose-histidine agar | Cycloheximide and nystatin (each at 25 µg/ml) | 14 days | 10 - 40 °C | Santhanam et al., 2012b |
|  | Minimal medium | Cycloheximide (50 µg/m) and nystatin (10 µg/ml) | 28 days | 28 °C | Saygin et al., 2020c |
|  | R2A agar | - | 7 days | 28 °C | Li, L. et al., 2019a |
|  | Raffinose-histidine agar |  | 14 days | 10 - 35 °C | Santhanam et al., 2012a |
|  | Tyrosine agar (ISP 7 medium) | K_2_Cr_2_O_7_ (25 mg/ml), calcium propionate (30 mg/ml) and cycloheximide (50 mg/ml) | 7 days | 20 - 40 °C | Li, Y. et al., 2018 |
|  | Glucose-yeast extract agar | Antifungal antibiotics and rifampicin (5 µg/ml) | 14 days | 10 - 37 °C | Santhanam et al., 2013 |
|  | Modified cellulose-casein-multi-salts medium (CCMS) + 10 N NaOH | - | 4 weeks | 30 °C | Zhang et al., 2013 |
|  | SCA medium | Nalidixic acid (10 µg/ml) and Amphotericin B (20 µg/ml) | 8 – 10 days | 28 °C | Nithya et al., 2017 |
|  |  | Actidione and nystatin (each at 25 µg/ml) | 21 days | 30 °C | Rateb et al., 2011a |
|  | Nutrient agar + 1% glucose | - | 10 days | 30 ± 2 °C | Abdelmoteleb and González-Mendoza, 2020 |
|  | Medium A |  | 14 days | 28 - 30 °C | Hozzein et al., 2011 |
|  | Gauze’s No. 1 agar | Nystatin (100 mg/ml) and nalidixic acid (50 mg/ml) | 21 days | 37 °C | Yuan et al., 2020 |
| *Streptosporangium* | Chitin-vitamin agar | Actidione (50 µg/ml) | - | 30 °C | Boubetra et al., 2016 |
|  |  | Polymyxin (25 mg/l) | 21 days | 25 - 37 °C | Chaouch et al., 2016 |
| *Tenggerimyces* | 1/5-strength R2A agar | - | 4 weeks | 28 - 30 °C | Sun et al., 2015 |
| *Williamsia* | R2A agar | - | 3 months | - | Guerrero et al., 2014 |
| *Yuhushiella* | Sauton’s agar | Cycloheximide and nystatin (each at 50 µg/ml) | 2 weeks | 37 - 45 °C | Mao et al., 2011 |
|  | Starch-casein agar | - | 2 weeks | 20 - 40 °C | Ibeyaima et al., 2016 |
